# Prognostic and therapeutic monitoring value of plasma and urinary cytokine profile in primary membranous nephropathy: the STARMEN trial cohort

**DOI:** 10.1093/ckj/sfae239

**Published:** 2024-08-12

**Authors:** Jorge Enrique Rojas-Rivera, Takehiro Hasegawa, Gema Fernandez-Juarez, Manuel Praga, Yuko Saruta, Beatriz Fernandez-Fernandez, Alberto Ortiz, the Sysmex R&D Center Europe team and STARMEN working group, Jesús Egido, Manuel Praga, Antolina Rodríguez-Moreno, Begoña Rivas, Mercedes Cao, Juan Ramón Gómez-Martino, Ana Ávila, José Bonet, Luis Quintana, Irene Agraz, Monserrat Diez-Encarnación, Cristina Rabasco, Virginia Cabello, Zhao Feng, Hanna Lunding, Dimitris Karalis, Yun Li

**Affiliations:** Department of Nephrology and Hypertension, IIS-Fundacion Jimenez Diaz UAM, Madrid, Spain; RICORS2040 Madrid, Spain; Sysmex R&D Center Europe, Hamburg, Germany; RICORS2040 Madrid, Spain; Department of Nephrology, HU La Paz, Madrid, Spain; Instituto de Investigación 12 de Octubre, Universidad Complutense de Madrid, Madrid, Spain; Sysmex R&D Center Europe, Hamburg, Germany; Department of Nephrology and Hypertension, IIS-Fundacion Jimenez Diaz UAM, Madrid, Spain; RICORS2040 Madrid, Spain; Departamento de Medicina, Facultad de Medicina, Universidad Autónoma de Madrid, Madrid, Spain; Department of Nephrology and Hypertension, IIS-Fundacion Jimenez Diaz UAM, Madrid, Spain; RICORS2040 Madrid, Spain; Departamento de Medicina, Facultad de Medicina, Universidad Autónoma de Madrid, Madrid, Spain

**Keywords:** anti-PLA2R, clinical trial, inflammation, membranous nephropathy, treatment

## Abstract

**Background:**

Primary membranous nephropathy (PMN) is usually caused by anti-phospholipase A2 receptor (PLA2R) autoantibodies. There are different therapeutic options according to baseline risk. Novel biomarkers are needed to optimize risk stratification and predict and monitor the response to therapy, as proteinuria responses may be delayed. We hypothesized that plasma or urinary cytokines may provide insights into the course and response to therapy in PMN.

**Methods:**

Overall, 192 data points from 34 participants in the STARMEN trial (NCT01955187), randomized to tacrolimus–rituximab (TAC-RTX) or corticosteroids–cyclophosphamide (GC-CYC), were analysed for plasma and urine cytokines using a highly sensitive chemiluminescence immunoassay providing a high-throughput multiplex analysis.

**Results:**

Baseline (pretreatment) urinary C-X-C motif chemokine ligand 13 (CXCL13) predicted the therapeutic response to TAC-RTX. Cytokine levels evolved over the course of therapy. The levels of nine plasma and six urinary cytokines correlated with analytical parameters of kidney damage and disease activity, such as proteinuria, estimated glomerular filtration rate and circulating anti-PLA2R levels. The correlation with these parameters was most consistent for plasma and urinary growth differentiation factor 15 (GDF15), plasma tumour necrosis factor α and urinary TNF-like weak inducer of apoptosis. Decreasing plasma GDF15 levels were associated with response to GC-CYC. Four clusters of cytokines were associated with different stages of response to therapy in the full cohort, with the less inflammatory cluster associated with remission.

**Conclusion:**

PMN displayed characteristic plasma and urine cytokine patterns that evolved over time as patients responded to therapy. Baseline urinary CXCL13 concentration could be a prognostic marker of response to TAC-RTX.

## Graphical Abstract

**Graphical Abstract:**
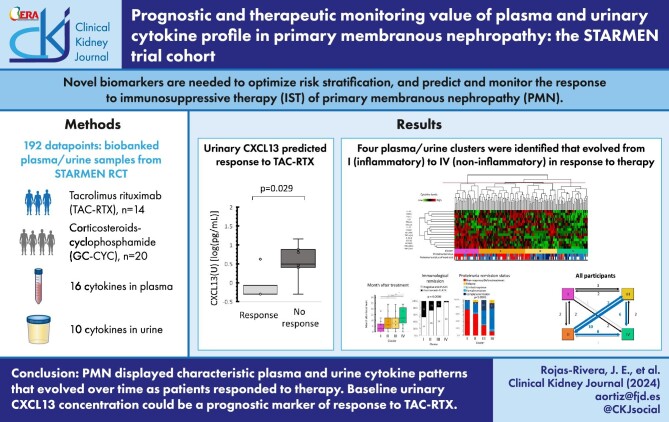

KEY LEARNING POINTS **What was known:**

- Primary membranous nephropathy (PMN) is a cause of kidney failure.
- There are different therapeutic options for PMN according to baseline risk.
- Several immunosuppressive therapy (IST) regimens are available, but markers to predict the efficacy of specific IST regimens are lacking.

**This study adds:**

- PMN is characterized by a pro-inflammatory cytokine phenotype in plasma and urine that evolves over time in response to IST.
- Baseline urinary C-X-C motif chemokine ligand 13 predicted the therapeutic response to tacrolimus–rituximab.
- Four clusters of cytokines were associated with different stages of response to therapy in the full cohort, with the less inflammatory cluster associated with remission.

**Potential impact:**

- The concept of cytokine response may complement the current concepts of immunological (i.e. disappearance of circulating anti-PLA2R antibodies) and proteinuria response to guide therapy for PMN.
- In this regard, the cytokine testing technology is ready for clinical implementation in a high-throughput manner.

## INTRODUCTION

Glomerular diseases are the most common cause of prevalent kidney replacement therapy (KRT) in Europe, having a negative impact on quality of life, life expectancy and healthcare costs [1, 2]. Primary membranous nephropathy (PMN) is an autoimmune disease, usually caused by anti-phospholipase A2 receptor (PLA2R) antibodies against podocytes that cause proteinuria and loss of kidney function [3–5]. PMN is the leading cause of nephrotic syndrome in non-diabetic Caucasian adults [3–5]. In Europe, PMN was the reason for >3000 cases of KRT in 2000–2019, illustrating the need for better management approaches [2].

The Kidney Disease: Improving Global Outcomes (KDIGO) 2021 guidelines for the management of glomerular diseases indicate that treatment and monitoring of patients with PMN depend on the baseline risk of progression, which is mainly based on proteinuria, kidney function and anti-PLA2R antibodies [1]. Around 70% of low-risk PMN patients fail to achieve spontaneous remission with antiproteinuric conservative therapy alone [5, 6]. Patients at moderate or high risk, as assessed by the 6-month response to antiproteinuric therapy, or at very high risk, should start immunosuppressive therapy (IST) [1, 7]. These criteria may delay the initiation of IST by at least 6 months. Several IST regimens are available, but markers to predict the efficacy of specific IST regimens are lacking. A trial-and-error approach is suggested by the KDIGO, in which a first IST regimen may be switched to another if, after 6 months of therapy, anti-PLA2R antibodies are still detectable, although some centres measure anti-PLA2R antibodies at month 3 and adapt treatment at that time [1]. Thus the current KDIGO strategy for treating PMN may delay initiation of an effective therapy for up to a year (the 6-month time needed to assign a high risk, initiate IST and the up to 6 months needed to eventually declare failure of the initial IST strategy). During this time, patients are exposed to risks derived from potentially lethal complications of nephrotic syndrome or IST. There is a clear need for biomarkers that predict the requirement for IST and the response to individual IST regimens at earlier time points, thus allowing earlier successful treatment that shortens disease duration. Of note, in different clinical trials, a complete or partial proteinuria response was only observed in 35–75% (6 months) and 50–80% of patients (12 months) [8], despite an immunological (disappearance of circulating anti-PLA2R antibodies) response of 70–92% at 6 months, again emphasizing the need for additional biomarkers that predict earlier the response or failure of therapy.

Although PMN is considered oligo-inflammatory (no glomerular leukocyte infiltration), mediators of inflammation, including cytokines and chemokines, are involved in its pathogenesis [9–11]. We hypothesize that the plasma and urinary cytokine profile may provide additional information on disease activity, from pathogenic events facilitating autoantibody production to tubulointerstitial inflammation to the systemic response to kidney injury, and may change in response to therapy. Thus, assessing baseline and post-treatment cytokine profiles may help in risk stratification and monitoring the response to IST, contributing to optimization of the choice of IST, timing of initiation or modification of IST, avoiding prolonged untreated periods or unnecessarily prolonging failed IST regimens, potentially increasing the efficacy and safety of interventions. Combining biochemical, antibody and cytokine profiles associated with PMN activity and its response to therapy is aligned with the concept of precision medicine.

As a first step towards that goal, we performed an exploratory analysis of the baseline cytokine profile in participants in a PMN clinical trial and its response to IST, exploring their value in predicting response to IST regimens and monitoring the inflammatory response and disease activity, and thus in guiding therapy. For this, we tested biobanked samples from STARMEN (NCT01955187) [12, 13], a randomized controlled trial comparing sequential tacrolimus and rituximab (TAC-RTX) versus alternating glucocorticoids and cyclophosphamide (GC-CYC) in PMN.

## MATERIALS AND METHODS

### Participants and study design

This was a post hoc analysis of the STARMEN clinical trial that randomized 86 patients with PMN and nephrotic syndrome persisting after 6 months of non-specific antiproteinuric therapy with GC-CYC (*n* = 43) or sequential treatment with TAC-RTX (*n* = 43) and prospectively collected biobanked plasma and urine samples [12, 13] (see Supplementary Methods).

### Cytokine assessment

Most plasma and urine levels of cytokines were measured using a fully automated immune analyser (HISCL-5000 or HISCL-800; Sysmex, Hyogo, Japan). Interleukin-17 (IL-17) levels were measured using a fully automated highly sensitive immune analyser (HI-1000; Sysmex). GDF15 levels were measured using the Human GDF15 Quantikine ELISA Kit (DGD150; R&D Systems, Minneapolis, MN, USA).

### Statistical analysis

Two-tailed *P*-values <.05 were considered significant. Fisher's exact test, Steel–Dwass test and Mann–Whitney U test were applied using R (R Foundation for Statistical Computing, version 4.0.3, Vienna, Austria). Exploratory analysis used Spearman's rank correlation between plasma and urine cytokine levels and various laboratory parameters, using each time point for the same patient as independent data. Unsupervised hierarchical cluster analysis was performed using Cluster 3.0 (Human Genome Centre, University of Tokyo, Tokyo, Japan), treating values of each time point for the same patient as independent data. Cytokines were selected for cluster analysis based on their correlation with kidney function, proteinuria and anti-PLA2R levels. For prediction of response to therapy, the STARMEN clinical trial definition of responder or non-responder was used, as assessed at the end of therapy in the primary analysis and at up to 24 months of follow-up in a sensitivity analysis. For analysis relating cytokine levels to current or future status of the patients, the active (pre-treatment, no response and relapse) and remission (limited, partial and complete remission) states at those specific time points were used. The cut-off cytokine levels were defined using the Youden index, based on all samples.

## RESULTS

### Demographic characteristics of the study population

A total of 192 biobanked blood and urine samples were available for cytokine studies from 34 STARMEN participants (22 men, 12 women; 14 TAC-RTX and 20 GC-CYC) and all were studied (Table 1). Demographic and clinical characteristics were similar to those of the full STARMEN population (Supplementary Table S1). At baseline, kidney function [median estimated glomerular filtration rate (eGFR) >80 ml/min/1.73 m^2^) was relatively preserved, proteinuria was nephrotic (median >7 g/24 h) and the prevalence of circulating anti-PLA2R antibodies was 71% (GC-CYC arm) and 56% (TAC-RTX). Almost all patients were on antiproteinuric therapy. Patients in the GC-CYC arm displayed a lower prevalence of no response and a higher prevalence of complete remission at 24 months [13].

**Table 1:** Demographics and clinical characteristics.

Characteristics	TAC-RTX (*n* = 16)		GC-CYC (*n* = 21)		*P*-value	
Age (years), median (IQR)	50.9 (47.5–54.8)		57.1 (48.5–62.3)		0.22	
Male, *n* (%)	13(81.3)		12(57.1)		0.166	
eGFR (ml/min/1.73 m^2^), median (IQR)	90.4 (64.2–104)		80.7 (57–94.2)		0.52	
Protein urine (g/24 h), median (IQR)	7.1 (5.4–10.5)		8.9 (6.2–11.9)		0.5	
Positive serum anti-PLA2R, *n* (%)	9 (56.3)		15 (71.4)		0.489	
Serum anti-PLA2R antibody titre (RU/ml), median (IQR)	24.2 (1.1–119.3)		42.1 (10.8–139.4)		0.434	
RASi, *n* (%)	16 (100)		20 (95.2)		1	
Diuretics, *n* (%)	13 (81.3)		16 (76.2)		0.68	
Systolic blood pressure (mmHg), median (IQR)	125.5 (121.5–128.5)		128 (113–149)		0.602	
Diastolic blood pressure (mmHg), median (IQR)	77 (71–82.5)		72 (69–80)		0.349	
Serum albumin (g/dl), median (IQR)	2.7 (2.4–2.9)		2.6 (2.4–2.9)		0.513	
Statins, *n* (%)	14 (87.5)		18 (85.7)		1	
Outcome, *n* (%)					After treatment	Pool 24 months
Relapse (after treatment, pool 24 months)	0 (0)	1 (6.3)	0 (0)	1 (4.8)	1.000	1.000
No response (after treatment, pool 24 months)	9 (56.3)	9 (56.3)	5 (23.8)	2 (9.5)	0.035	0.002
Limited response (after treatment, pool 24 months)	2 (12.5)	0 (0)	0 (0)	0 (0)	0.162	1.000
Partial remission, (after treatment, pool 24 months)	4 (25)	5 (31.3)	12 (57.1)	5 (23.8)	0.092	0.704
Complete remission (after treatment, pool 24 months)	1 (6.3)	1 (6.3)	4 (19)	13 (61.9)	0.379	0.001

IQR: interquartile range; RASi: renin–angiotensin system inhibitor.

*P*-values were calculated using the Mann-Whitney U test or Fisher’s exact test.

After treatment: outcome just after treatment protocol, 6-months for GC-CYC, 9-months for TAC-RTX; pool 24 months: final status at 24 months.

### Baseline cytokine levels and treatment response to specific IST regimens

First, we addressed whether baseline cytokine levels predicted the response to specific IST regimens. For that, pretreatment plasma or urine biomarker levels were compared with treatment outcomes (Supplementary Table S2 for TAC-RTX, Supplementary Table S3 for GC-CYC).

**Figure 1:**
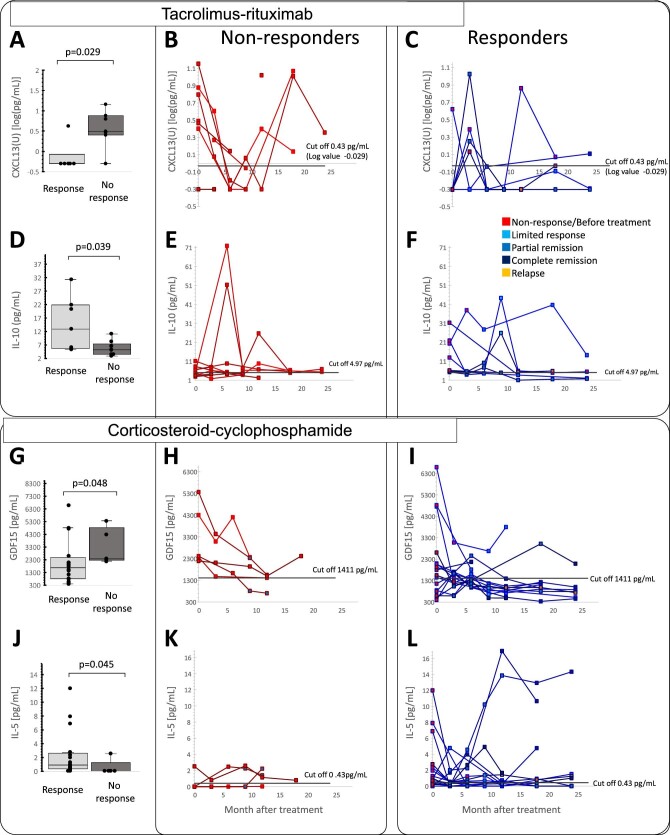
Baseline and follow-up biomarker levels and treatment outcomes. (**A–F)** TAC-RTX arm; (**G–L)** GC-CYC arm. (**A, D, G and J**) Baseline values for responders and non-responders for selected cytokines (urinary CXCL13, serum IL-10, serum GDF15 and serum IL-5). Results are shown as individual data points (circles) with medians (bars) and interquartile ranges (box). Whiskers extend to the minimum and maximum values excluding outliers. *P*-values were calculated using the Mann–Whitney U test. (**B, C, E, F, H, I, K, L)** Change in biomarker levels during treatment. Squares along the line indicate patient status at each point. Non-responder: non-response status at 9 months **(B, E)** and 6 months **(H, K)**; responder limited, partial or complete remission status at 9 months **(C, F)** and 6 months **(I, L)**. U: urine biomarker levels. Outcome defined at the end of treatment.

In the TAC-RTX arm, non-responders, as assessed at the end of treatment (9 months), exhibited significantly higher baseline (pretreatment) urinary C-X-C motif chemokine ligand 13 (CXCL13) and lower plasma IL-10 levels (Fig. 1A–F). Urinary CXCL13 levels were clearly higher in most non-responders than in responders (Fig. 1A–C) while for plasma IL-10 levels, there was overlap (Fig. 1D–F). The positive predictive value of baseline urinary CXCL13 for non-response was 86% and the negative predictive value was 83% (Supplementary Table S4, Supplementary Fig. S1). In a sensitivity analysis, response was evaluated up to 24 months (Supplementary Table S5). Low baseline urinary CXCL13 was associated with an eventual response in 100% of participants randomized to TAC-RTX while high baseline values were associated with no response or relapse at last follow-up in 100% of participants randomized to TAC-RTX.

Only five TAC-RTX patients were serum anti-PLA2R negative, which precludes formal statistical testing. However, one had urinary CXCL13 above the threshold and did not respond at either 9 or 24 months, and the other four had undetectable urinary CXCL13: 3 (75%) had either a partial response or complete response at the end of treatment (9 months) and all had a partial response or complete response at 24 months.

During TAC treatment, urinary CXCL13 levels decreased (Fig. 1B, C). Once TAC was stopped, urinary CXCL13 levels of non-responders returned to baseline levels, all becoming above the threshold again, while most responders remained below the threshold (Fig. 1B, Supplementary Table S.6).

**Figure 2:**
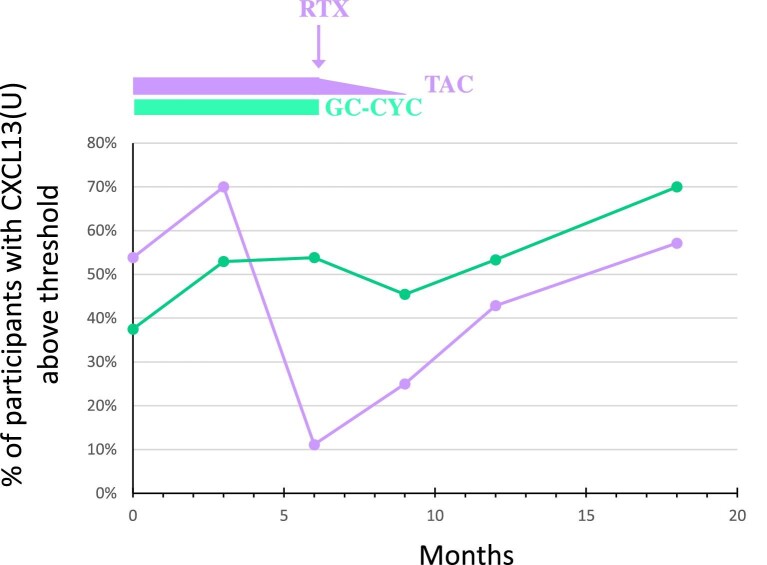
Percentage of patients in each treatment arm with urinary CXCL13 levels above the cut-off established for the TAC-RTX arm. All participants are shown for both treatment arms, including responders and non-responders.

In the GC-CYC arm, baseline urinary CXCL13 did not predict response (Supplementary Fig. S2) and urinary CXCL13 levels were less responsive to initiation of GC-CYC at early time points (up to 18 months) (Fig. 2, Supplementary Table S.6). Also, non-responders exhibited higher baseline plasma GDF15 and lower IL-5 levels (Fig. 1G, J). Plasma GDF15 levels in most non-responders remained above the cut-off levels, whereas they eventually dropped below the cut-off levels in responders (Fig. 1H, I). However, there was overlap in plasma GDF15 and IL-5 levels between responders and non-responders (Fig. 1H, I, K, L).

### Correlation between cytokine levels and clinical data

In exploratory analyses, some plasma and urine cytokine levels, measured at baseline or during follow-up, correlated with laboratory parameters related with the pathophysiology and evidence of kidney damage of PMN (Table 2). Plasma levels of IL-17, CXCL13, tumour necrosis factor α (TNF-α), TNF-like weak inducer of apoptosis (TWEAK) and GDF15 correlated with serum anti-PLA2R titres. In all cases, except for CXCL13, the correlation was positive. Additionally, plasma IL-8, CXCL9, TNF-α and GDF15 showed significant correlation with kidney damage assessed by decreased eGFR. Plasma IL-8, IL-10, IL-17, C-C motif chemokine ligand 20 (CCL20), TNF-α and GDF15 correlated with kidney damage assessed as the magnitude of proteinuria. Overall, plasma IL-8, TNF-α and GDF15 levels correlated with both lower eGFR and higher proteinuria.

**Table 2:** Correlation coefficients of the clinical and biological parameters.

	Serum anti-PLA2R antibody (RU/ml)		eGFR		Urine protein:creatinine ratio		Urine protein (g/24 h)		Serum creatinine (mg/dl)		Minimum
Biomarkers	*r*	*P*-value	*r*	*P*-value	*r*	*P*-value	*r*	*P*-value	*r*	*P*-value	*P*-value
IL-5	−0.06	.457	−0.03	.729	0.02	.805	0.06	.426	0.05	.553	.426
IL-6	−0.02	.804	−0.15	.052	0.12	.129	0.09	.246	0.1	.201	.052
IL-8	0.02	.849	−0.16	.046	0.18	.022	0.19	.015	0.13	.094	.015
IL-10	−0.1	.226	−0.04	.613	0.17	.032	0.12	.133	0.03	.683	.032
IL-17	0.21	.006	−0.14	.080	0.17	.036	0.15	.057	0.08	.300	.006
IL-18	−0.12	.142	−0.03	.706	−0.05	.537	−0.01	.930	0.09	.259	.142
IL-22	0.06	.474	−0.01	.899	0.12	.147	0.1	.203	0	.983	.147
IL-23	0.04	.582	−0.01	.881	0	.991	0.03	.742	0.03	.667	.582
CXCL9	0.08	.300	−0.16	.039	0.13	.111	0.04	.596	0.07	.397	.039
CXCL13	−0.21	.008	0.08	.292	−0.07	.388	−0.12	.147	−0.11	.161	.008
CCL5	0.03	.677	−0.03	.717	0.01	.917	−0.02	.763	−0.02	.811	.677
CCL17	−0.03	.669	−0.03	.754	−0.03	.759	−0.06	.427	−0.03	.674	.427
CCL20	0.13	.101	0.02	.791	0.19	.019	0.2	.010	−0.04	.629	.010
TNF-α	0.38	<.001	−0.36	<.001	0.47	<.001	0.48	<.001	0.36	<.001	<.001
TWEAK	0.16	.039	0	.969	0.1	.205	0.1	.200	−0.1	.184	.039
GDF15	0.17	.033	−0.52	<.001	0.5	<.001	0.45	<.001	0.46	<.001	<.001
IL-6 (U)	0.2	.014	−0.31	<.001	0.12	.157	0.17	.048	0.28	.001	<.001
IL-8 (U)	0.04	.649	0.03	.745	−0.08	.350	−0.07	.383	−0.15	.063	.350
IL-17 (U)	0.2	.014	−0.02	.823	0.17	.040	0.18	.031	−0.02	.777	.014
IL-18 (U)	0.27	.001	0.03	.766	0.3	<.001	0.37	<.001	−0.01	.924	<.001
IL-22 (U)	0.13	.109	0.04	.613	−0.03	.707	−0.06	.512	−0.02	.789	.109
CXCL13 (U)	−0.05	.540	−0.08	.340	0.03	.726	−0.07	.384	0.03	.732	.340
CCL17 (U)	−0.03	.679	−0.08	.339	−0.2	.019	−0.14	.087	0.04	.673	.019
TNF-α (U)	0.08	.349	0.08	.347	−0.18	.029	−0.13	.117	−0.16	.048	.029
TWEAK (U)	0.17	.036	−0.23	.004	0.29	<.001	0.31	<.001	0.27	.001	<.001
GDF15 (U)	0.25	.002	−0.22	.007	0.31	<.001	0.29	.001	0.18	.031	<.001

Correlation coefficients of the clinical parameters and biomarker levels in patients. All the available time series samples were used in the analysis. The correlation coefficients were generated by Spearman's correlation analysis. Results were considered significant at *P* < .05.

U: urine biomarker levels.

The levels of some urinary cytokines also exhibited significant correlation with laboratory parameters. Urine levels of IL-6, IL-17, IL-18, TWEAK and GDF15 correlated with serum anti-PLA2R titres. Additionally, urinary IL-6, TWEAK and GDF15 correlated with kidney damage assessed by decreased eGFR, while urinary IL-17, IL-18, TWEAK and GDF15 correlated with kidney damage assessed as the magnitude of proteinuria.

Overall, plasma TNF-α and GDF15 and urinary TWEAK and GDF15 most consistently correlated with more active disease and kidney damage assessed as either higher anti-PLA2R titres, lower eGFR or higher proteinuria.

### Cytokine phenotyping through cluster analysis

**Figure 3:**
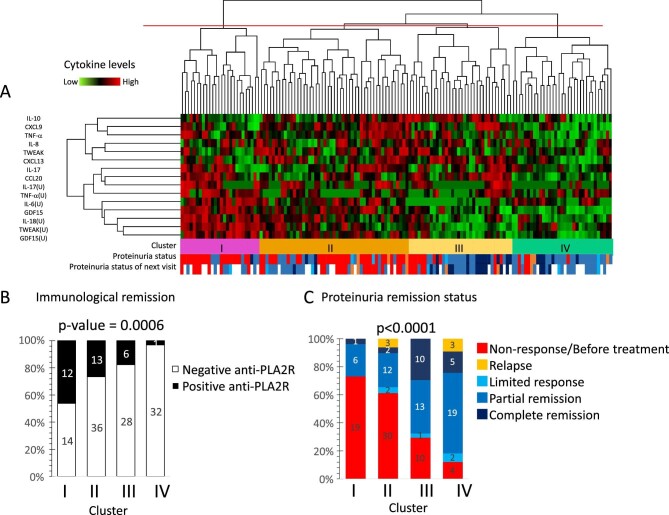
Unsupervised hierarchical clustering analysis of inflammatory cytokine levels and association with clinical features. The cluster analysis disclosed four global cytokine clusters. (**A)** Unsupervised hierarchical clustering analysis of inflammatory cytokine levels. Cluster analysis was performed by a complete linkage based on city block distance. Patient proteinuria status at the time of sample collection and next visit are indicated at the bottom of the cluster. (**B)** Immunological remission: white rectangle indicates immunological remission (negative anti-PLA2R). (**C)** Proteinuria remission status. The four identified clusters are indicated at the bottom of each graph, in the horizontal axis. The statistical significances between the clusters were calculated using the Fisher's exact test. U: urine biomarker levels. Outcome defined as patient condition at the time of sample collection.

To better understand heterogeneity in the inflammatory pathophysiology, inflammatory patterns and their relationship to pathological parameters were assessed using unsupervised hierarchical cluster analysis in the full cohort to develop global (i.e. using all samples available from both treatment arms) cytokine clusters. This disclosed four global cytokine phenotype clusters based on 15 cytokines (9 in plasma and 6 in urine) that showed significant correlation with any of the clinical parameters listed in Table 2 (Fig. 3A). Patients were classified into four clusters. The most striking differences were generally found between clusters 1 and 4 (Supplementary Fig. S3). In cluster 1, plasma TNF-α, CCL20 and GDF15, as well as all urine cytokines, were upregulated, indicating severe systemic and urinary inflammation. Cluster 2 differed from cluster 1 by showing higher plasma levels of the anti-inflammatory cytokine IL-10, while levels of the T helper 1 cytokine CXCL9 were also elevated compared with cluster 1. The levels of plasma CCL20 and four of six urine cytokines were significantly lower than those in cluster 1. In cluster 3, plasma IL-10 levels were also higher than those in cluster 1, and except for IL-17, urine cytokine levels were lower than those in cluster 1. Both cluster 2 and cluster 3 represented milder urinary inflammation, but systemic inflammation was sustained. The key difference between clusters 2 and 3 was the even milder urine inflammation in cluster 3 than in cluster 2, as well as higher plasma CCL20 levels. In cluster 4, all cytokines were downregulated (non-inflammatory phenotype). Plasma and urine GDF15 progressively decreased from cluster 1 to 4 and displayed, together with plasma CCL20 and urine IL-18, the highest number of statistically significant differences between clusters.

The global cytokine clusters provided information on the evolution of PMN in response to treatment. Sampling points were earlier in time for cluster 1 than for clusters 3 and 4, and earlier for clusters 2 and 3 than for cluster 4 (Supplementary Fig. S4A). The prevalence of serum anti-PLA2R positivity and anti-PLA2R levels (Fig. 3B, Supplementary Fig. S4B) and values of eGFR (Supplementary Fig. S4C), 24-h urinary protein excretion (Supplementary Fig. S4D) and urine protein:creatinine ratio (Supplementary Fig. S4E) were consistent with more active disease in cluster 1 and had significantly improved in cluster 4 compared with cluster 1. In this regard, most (73%) samples in cluster 1 corresponded to pretreatment time points or to time points in which patients had not responded to therapy, whereas most (78%) samples in cluster 4 were in partial or complete remission, i.e. had improved as compared with baseline in response to therapy (Fig. 3C). A majority (67%) of samples in cluster 2 corresponded to non-responsive or relapse patients (Fig. 3C).

**Figure 4:**
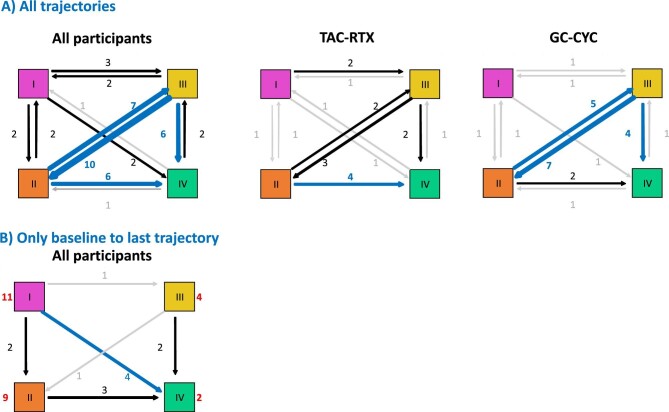
Transition of cytokine clusters. (**A)** Cluster trajectories between two consecutive sampling points. The number represents the number of sample pairs with that trajectory. Trajectories with four or more sample pairs are represented in blue and those with only one in grey. (**B)** Cluster trajectories from baseline to the latest sampling point. The number of patients at baseline is indicated next to each cluster in red characters.

Analysis of cluster trajectories was also consistent with a gradient of activity between cluster 1, clusters 2 and 3 and cluster 4 (Fig. 4A). There was frequent exchange between clusters 2 and 3 and progression from clusters 2 and 3 to 4, while direct transition from cluster 1 to 4 was uncommon. Baseline to last observation trajectories (Fig. 4B), as well as individual patient trajectories (Supplementary Fig. S5), are also consistent, suggesting that achieving cluster 4 status may be considered as the aim of therapy, as it is associated with treatment response. In this regard, ≈50% of participants reached cluster 4 at months 3–6 or 9.

### Cytokine phenotyping through cluster analysis in treatment arms

Applying the four global clusters to each treatment arm disclosed overall concordance in the two arms between cluster 4 and immunological or proteinuria response to therapy, as well as a higher prevalence of cluster 4 in the GC-CFM arm, which is consistent with the better response to therapy in this arm (Supplementary Fig. S6). However, both treatment arms differed markedly in the association of cluster 3 with outcomes: cluster 3 was common in the TAC-RTX arm, where it was not clearly associated with current or future response to therapy, and more uncommon in the GC-CFM arm, where it was associated with immunological response to therapy and current and, above all, future proteinuria response.

**Figure 5:**
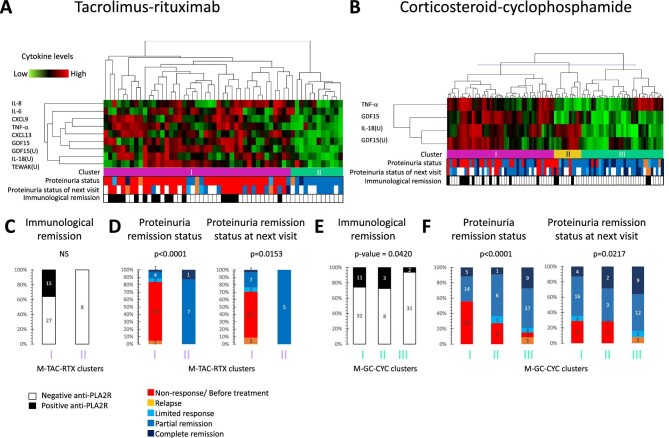
Treatment-specific monitoring cytokine clusters for disease activity. TAC-RTX group **(A, C, D)** or GC-CYC group **(B, E, F)**. (**A, B)** Unsupervised hierarchical clustering analysis of cytokines, which were selected based on volcano plots, yielded two clusters for disease monitoring in the TAC-RTX arm (M-TAC-RTX clusters 1 and 2: labels coloured purple in panels C and D) and three in the GC-CYC arm (M-GC-CYC clusters 1, 2 and 3: labels coloured in burgundy E and F). The cluster analysis was performed by average linkage based on Euclidean distance. Patient proteinuria status at the sample collection and next visit and status of immunological remission are indicated. (**C, E)** Immunological status. (**D, F)** Proteinuria remission status in the clusters. Statistical significances calculated using the Steel–Dwass test or Fisher's exact test. U: urine biomarker levels. Outcome is defined as the patient condition at the time of sampling.

Given these differences between treatment arms, we next used a volcano plot to select plasma and urine biomarkers for monitoring the therapeutic response through individualized clusters for the TAC-RTX and GC-CYC arms, acknowledging the distinct modes of action of these immunosuppressants (Supplementary Fig. S7A, B). We selected biomarkers whose median levels were significantly reduced (≤0.75 times) in the remission condition. Plasma GDF15 and TNF-α as well as urine GDF15 and IL-18 were shared as cluster components by both treatment arms, while the TAC-RTX arm clusters also contained plasma CXCL13 and other cytokines (Fig. 5, Supplementary Fig. S7).

Cluster analysis based on these biomarkers classified samples into two novel clusters for the TAC-RTX arm and three clusters for the GC-CYC arm, in both cases using a lower number of cytokines than the global clusters. The new clusters were labelled M- (for monitoring) TAC-RTX (Fig. 5A) or GC-CYC (Fig. 5B). Cluster M-TAC-RTX-2 or M-GC-CYC-3 displayed low levels of all selected biomarkers (Supplementary Fig. S7C, D) and were dominated by samples from patients that had the lowest anti-PLA2R titres (Supplementary Fig. S7E, F) and were almost all in immunological remission (Fig. 5C, E) and partial or complete remission status (all samples in cluster M-TAC-RTX-2 and 79% of samples in cluster M-GC-CYC-3) (Fig. 5D, F). M-GC-CYC-3 displayed only upregulated urine IL-18 and GDF-15. Patients represented in clusters M-TAC-RTX-2 and M-GC-CYC-3 sustained their proteinuria remission status when assessed 3–6 months later. In contrast, most samples from M-TAC-RTX-1 corresponded to baseline or no proteinuria response. The two M-TAC-RTX clusters separated immunological/proteinuria remission status more clearly than the three M-GC-CYC clusters.

## DISCUSSION

The main finding is that PMN is characterized by an inflammatory phenotype that can be monitored by assessing plasma and urine cytokines that may provide information on the future and ongoing response to therapy. Specifically, baseline urinary levels of the B cell homing cytokine CXCL13 [14, 15] predicted the future response to TAC-RTX, so it may contribute to decisions on the IST regimen in moderate- or high-risk patients. Plasma or urine cytokine levels have the potential to detect inflammation that is not apparent using current routine clinical or biochemical methods and four plasma/urine cytokine clusters were associated with kidney damage and response to therapy. The cytokine clusters evolved in response to therapy from pro-inflammatory cluster 1 to non-inflammatory cluster 4, which was related to serological and clinical response to treatment.

Circulating anti-PLA2R antibodies allow the diagnosis of PMN, provide prognostic information and allow monitoring the immunological response to therapy with changes in serum generally preceding changes in proteinuria [7, 16–22]. However, anti-PLA2R antibodies may be absent and do not predict response to specific IST regimens. Additionally, there may be mismatches between immunological (70–92% at 6 months) and proteinuria responses (50–80% at 12 months in STARMEN) [8]. Cytokine biomarkers should be considered an addition to the current biomarker armamentarium (anti-PLA2R, GFR, proteinuria). Furthermore, cytokine profiles may be informative prior to treatment initiation (e.g. baseline urinary CXCL13 and future response to TAC-RTX) and earlier during treatment than current routine biomarkers. In this regard, despite the low number of TAC-RTX participants with negative baseline anti-PLA2R antibodies, their response to TAC-RTX was aligned with findings in anti-PLA2R-positive participants.

Plasma and urinary CXCL13 levels were associated with the proteinuria response to TAC-RTX. Moreover, urinary CXCL13 decreased in response to tacrolimus while patients were on this medication. CXCL13 and its receptor CXCR5 control the organization of B cells within follicles in lymphoid tissues [14, 15]. CXCL13 from T peripheral helper (Tph) cells activates CXCR5-expressing T follicular helper (Tfh) cells and B cells. Thus CXCL13 controls the segregation of lymphocytes between T and B cell compartments and modulates autoimmunity as it drives germinal centre B cells to the light zone where antigen selection occurs [23–25]. Tacrolimus inhibits Tfh cell differentiation and Tfh-mediated B cell activation and immunoglobin G (IgG) antibody secretion [26, 27]. In fact, tacrolimus specifically suppresses both lymph node and circulating Tfh cells, despite not impacting their regulatory counterparts or other CD4 T cell subsets [28]. The molecular mechanisms of this selectivity remain poorly understood, but the present report suggests a potential impact on CXCL13 that should be characterized in detail. In this regard, the decrease in urinary CXCL13 while on tacrolimus and the subsequent increase upon tacrolimus withdrawal is aligned with the clinical observation that tacrolimus monotherapy is associated with a high relapse rate [1]. Rituximab was also reported to reduce circulating CXCL13 levels [29, 30]. CXCL13 is expressed in injured kidneys, suggesting a kidney origin for urinary CXCL13, and may be pathogenic in antibody-mediated nephropathies [31–35]. Indeed, plasma CXCL13 was higher in patients with systemic lupus erythematosus and lupus nephritis [36] and in patients with anti-neutrophil cytoplasmic antibody vasculitis with active disease [37] while urinary CXCL13 was increased in antibody-mediated kidney graft rejection [38]. CXCL13 may directly activate podocytes to secrete pro-inflammatory mediators [39]. However, these potential pathomechanisms remain speculative, as a potential role of CXCL13 in kidney injury can only be addressed by clinical trials specifically targeting this chemokine.

Other cytokines are associated with PMN activity and kidney damage and have been previously shown to have key roles in kidney injury, including PMN. Thus TWEAK increases PLA2R expression in human podocytes, a response prevented by tacrolimus [11], while IL-17 drives the formation of kidney tertiary lymphoid organs in primary glomerular disease, a feature associated with high circulating CXCL13 levels [40]. Plasma GDF15 and TNF-α and urinary TWEAK and GDF15 most consistently correlated with more active disease or more kidney damage assessed as either higher anti-PLA2R titres, lower eGFR or higher proteinuria. Urinary TWEAK and GDF15 may reflect local availability of these cytokines and likely local production. Thus urinary GDF15 highly correlated with kidney biopsy GDF15 expression in a dataset that included PMN [41]. In this regard, since there is evidence for local kidney production of urinary cytokines, no adjustment was made for urinary creatinine, a marker of glomerular filtration. A similar approach is used in clinical practice for urinary markers of kidney injury thought to be produced locally (e.g. urinary neutrophil gelatinase-associated lipocalin, tissue inhibitor metalloproteinase-2/insulin-like growth factor binding protein 7) [42] and for other biomarkers of risk in PMN (e.g. urinary IgG, β2-microglobuin, α1-microglobulin [1]. In this regard, most participants with low baseline urinary CXCL13 had undetectable levels, so this decision did not modify these specific results.

Simultaneous assessment of multiple biomarkers may overcome limitations of individual biomarkers regarding their modification by alternative triggers [43]. In this regard, four global cytokine phenotype clusters were related to other biomarkers of the PMN response to therapy, such as serum anti-PLA2R antibodies and proteinuria, but there was only partial overlap with these biomarkers, suggesting that cytokine clusters provide additional information in an anti-PLA2R agnostic manner, highlighting their potential contribution to a more holistic evaluation of disease activity. However, larger studies are needed to define their precise role, if any, in the follow-up of PMN. Cluster 1 reflected systemic and local inflammation as well as disease activity and kidney damage, clusters 2 and 3 reflected interchangeable intermediate states and cluster 4 represented inactive disease and patients in cluster 4 who had not yet achieved remission did so in early follow-up time points.

**Figure 6:**
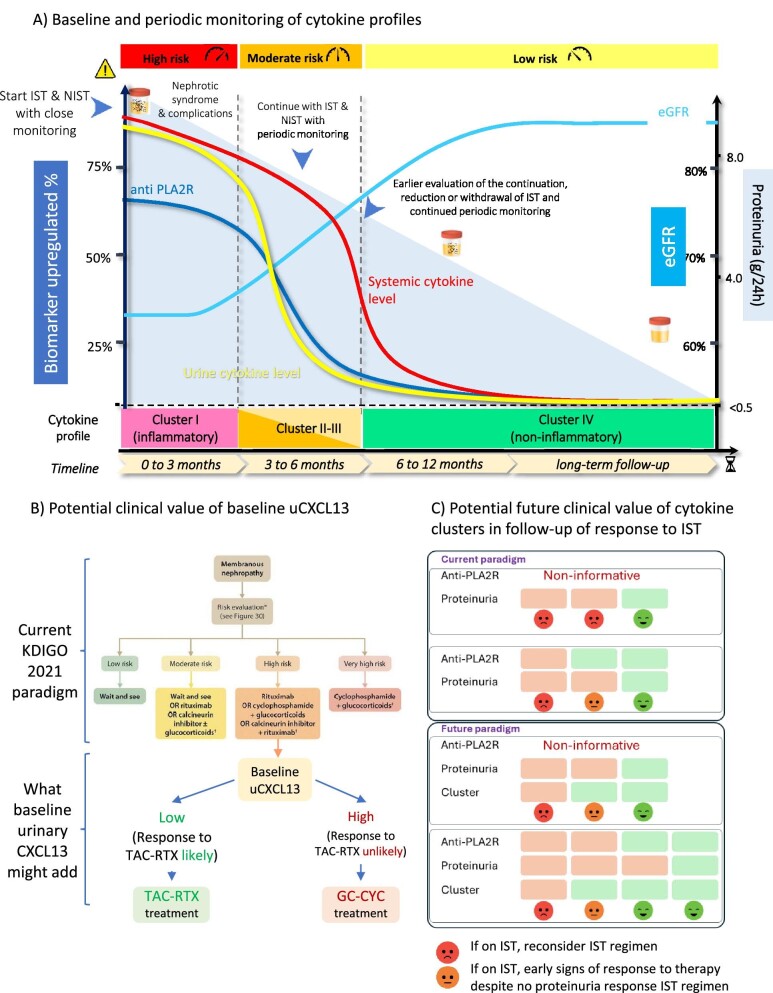
Precision approach to monitoring and treatment of membranous nephropathy: role of cytokine profiles. (**A)** Baseline and periodic monitoring of cytokine profiles could contribute to the early identification of patients who will respond to specific IST regimens. For responding patients, the baseline inflammatory profile of cytokines (cluster 1) will evolve to less inflammatory (clusters 2 and 3) or non-inflammatory (cluster 4) profiles. For patients predicted to not respond or not responding in periodic monitoring, alternative therapeutic approaches should be considered. This additional information may contribute to shortening the times for therapeutic decision-making. The figure is a conceptual representation of data from Fig. 3. NIST: non-immunosuppressive therapy (e.g. renin–angiotensin system blockers or other antiproteinuric therapies). (**B)** Context of use 1. After deciding to prescribe IST because of a KDIGO 2021 guideline high risk, urinary CXCL13 may be incorporated into decision-making about the optimal IST regimen by adding information of the chances of response to TAC-RTX. In this regard, the KDIGO 2021 guideline states that ‘Rituximab and CNIs have fewer and milder side effects than cyclophosphamide. Therefore, most physicians and patients will prefer initial treatment with rituximab or CNIs over treatment with cyclophosphamide.’ Thus, urinary CXCL13 may be useful in clinical decision-making at this point to allow gauging the probability of response to TAC-RTX. (**C)** Context of use 2. Conceptual representation of the potential added value of cytokine clusters when integrated with anti-PLA2R titres and proteinuria values in the follow-up of the response to IST. The precise role of cytokine clusters in follow-up is less well defined at this point than the potential role of baseline urinary CXCL13, but baseline evaluation and follow-up of cytokine clusters may provide added information that may be integrated with that provided by anti-PLA2R levels (in those patients who had positive baseline values) and proteinuria responses for a more holistic follow-up, which may signal earlier a positive response to IST than proteinuria alone. In the future, following further validation in anti-PLAR2-negative PMN, the simultaneous lack of response of proteinuria and cytokines may lead to reconsideration of the IST regimen earlier than current practice. Each column of coloured squares in panel B represents a potential follow-up combination of biomarkers and the putative interpretation. Both the contexts of use represented in panels B and C require external validation.

Overall, cytokine assessment provided more information in the TAC-RTX than the GC-CYC arm. The different effectiveness and duration of therapy and the different mechanism of action may contribute to this observation. The lower response rate in the TAC-RTX arm may have facilitated the finding of predictors of response and non-response, a more difficult task when an overwhelming majority of participants responded, as for GC-CYC. In this regard, the M-TAC-RTX-2 cluster, specific for TAC-RTX, was associated with both current and future response in all cases. Given the high proteinuria response rate, the present study was less well suited to explore biomarkers of treatment resistance in the GC-CYC arm. However, cytokine phenotyping did provide information on the response to TAC-RTX, an IST regimen considered less aggressive and safer by some authors [1, 7, 8]. Confirmation of the present findings in other clinical trials may support an approach of initial tacrolimus, rituximab or a mixed TAC-RTX regimen in patients at high risk with low CXCL13 levels (Fig. 6) who may be switched to GC-CYC if cytokine cluster analysis suggests no response without the need to wait for up to 24 months for a proteinuria response. Regarding stratification of risk as high risk, the KDIGO 2021 guidelines list up to eight biomarkers that may be informative, ranging from eGFR to urinary α1-microglobulin [1], illustrating how this is still an unmet medical need. Future studies should address whether cytokine clusters could add information in this regard by directly comparing performance to these biomarkers.

Several limitations should be acknowledged. As a rare disease, the number of participants was low, as for most PMN trials, which may result in potential imbalances [8]. Although both arms were not compared head to head in the present analysis, any imbalance may have influenced the results, as is the case for the percentage of participants with anti-PLA2R antibodies. The small number of participants also precluded meaningful subgroup analysis (e.g. by baseline eGFR or anti-PLA2R levels). The design of the study did not allow us to explore cytokine biomarkers to predict spontaneous remission in IST-naïve patients. Thus the study population (participants in a clinical trial) may not represent the full spectrum of the PMN population, as they already met high-risk criteria for initiating IST. Future studies should focus on samples from patients presenting with PMN before completing the initial 6 months of antiproteinuric therapy. Additionally, neither tacrolimus nor rituximab monotherapy was explored. Among the strengths, samples were prospectively collected in a randomized controlled trial and the study population was representative of the clinical trial population. Moreover, the unbiased approach testing multiple cytokines allowed us to build cytokine clusters that may overcome the shortcomings of individual cytokines. Finally, the testing technology is ready for clinical implementation in a high-throughput manner that would allow reference laboratories to offer the resource while limiting costs.

In conclusion, PMN displayed characteristic plasma and urine cytokine patterns that evolved over time as patients responded to therapy and may contribute to monitoring the therapeutic response (Fig. 6). Specifically, baseline urinary CXCL13 concentration differed between TAC-RTX responders and non-responders and could predict response to TAC-RTX. Given that the KDIGO 2021 guidelines state: ‘RTX and CNIs have fewer and milder side effects than CYC. Therefore, most physicians and patients will prefer initial treatment with RTX or CNIs over treatment with CYC’, baseline urinary CXCL13, representing the chances for success of a TAC-RTX strategy, may be incorporated into clinical decision-making (Fig. 6B). The path for clinical application of cytokine clusters is less straightforward and would require more extensive validation of their added value when integrated with anti-PLA2R titres and proteinuria values in the follow-up of the response to IST, as illustrated conceptually in Fig. 6C. Overall, these promising results, obtained in a limited number (*n* = 13) of participants, should be validated in larger cohorts that should also explore the contribution of cytokine assessment to the initial prediction of disease evolution at the time of diagnosis. These studies should further develop the concept of cytokine response that may complement the current concepts of immunological (i.e. disappearance of circulating anti-PLA2R antibodies) and proteinuria response.

## Supplementary Material

sfae239_Supplemental_Files

## Data Availability

The data underlying this article cannot be shared publicly due to ethical regulations. The data will be shared upon reasonable request to the corresponding author.
