# Supplementary material for: Prognostic and therapeutic monitoring value of plasma and urinary cytokine profile in primary membranous nephropathy: the STARMEN trial cohort
Source: Clin Kidney J. 2024 Aug 12;17(8):sfae239. doi: 10.1093/ckj/sfae239 (PMC11345640; doi:10.1093/ckj/sfae239)
Supplement: sfae239_Supplemental_Files [file sfae239_supplemental_files.zip › 70 Supplementary_methods_D20_R1.docx]

**Prognostic and therapeutic monitoring value of plasma and urinary cytokine profile in primary membranous nephropathy: the STARMEN trial cohort**

**Supplementary methods**

**Participants and study design**

STARMEN clinical trial (NCT01955187) randomized 86 patients with PMN and nephrotic syndrome persisting after six months of nonspecific antiproteinuric therapy to six-month cyclical treatment with corticosteroid and cyclophosphamide (GC-CYC, n=43) or sequential treatment with tacrolimus (full-dose for six months and tapering for another three months for a total to 9 months) and rituximab (1g at month six) (TAC-RTX, n=43)/suppl methods). It was performed in several hospitals in Spain and the Netherlands. The median time from kidney biopsy to enrollment was 6.2 months, but it was 18 months or longer in 25% of participants. The primary outcome of complete or partial remission of nephrotic syndrome at 24 months occurred in 36 patients (83.7%) in the GC-CYC arm and in 25 patients (58.1%) in the TAC-RTX arm. Complete remission at 24 months occurred in 26 patients (60%) in the GC-CYC arm and in 11 patients (26%) in the TAC-RTX arm. Anti-PLA2R titers decreased in both groups. The proportion of anti-PLA2R-positive patients who achieved immunological response (depletion of anti-PLA2R antibodies) at three and six months in the GC-CYC arm was 77% and 92%, respectively and in the TAC-RTX arm 45% and 70%, respectively. A total of 34 patients (14 from the TAC-RTX arm and 20 from the GC-CYC arm) had biobanked samples at baseline and during follow-up that were tested in the present study. The study complied with the requirements of the Declaration of Helsinki and was approved by the Medical Research Ethics Committee of all participating centers. Written informed consent was obtained from all patients. Exclusion criteria included previous treatment with corticosteroids in the three months period before screening, or previous treatment with other immunosuppressive agents in the six months period before screening, as well as previous treatment with rituximab or any other biological agent in the two years period before screening.

**Definitions of therapeutic response**

The following pre-specified definitions were used: Complete remission: a reduction of proteinuria to ≤0.3 g/24-hours plus stable kidney function (estimated glomerular filtration rate, eGFR ≥45 mL/min/1.73 m^2^). Partial remission: a reduction of proteinuria to 0.3–3.5 g/24-hours and 50% lower than baseline with stable kidney function (eGFR ≥45 mL/min/1.73 m^2^). Limited response: a reduction of proteinuria >50% from baseline levels, but to a value > 3.5 g/24-hours. Relapse: reappearance of proteinuria >3.5 g/24-hours and at least 50% higher than the lowest posttreatment value in at least three consecutive visits in those who previously presented a partial or complete remission. Immunological response: A level of anti-PLA2R ≤14 RU/mL in patients positive for baseline anti-PLA2R. No response: A reduction of proteinuria <50% from baseline level. Non-responders: patients fulfilling criteria for no response or limited response at 24 months as well as those that relapsed or switched to another immunosuppressive treatment other than the one assigned at any time during the follow-up.
