# Supplementary material for: Prognostic and therapeutic monitoring value of plasma and urinary cytokine profile in primary membranous nephropathy: the STARMEN trial cohort
Source: Clin Kidney J. 2024 Aug 12;17(8):sfae239. doi: 10.1093/ckj/sfae239 (PMC11345640; doi:10.1093/ckj/sfae239)
Supplement: sfae239_Supplemental_Files [file sfae239_supplemental_files.zip › Supplementary NDT update 20240226 R1 hase A2.pptx]

## Slide 1
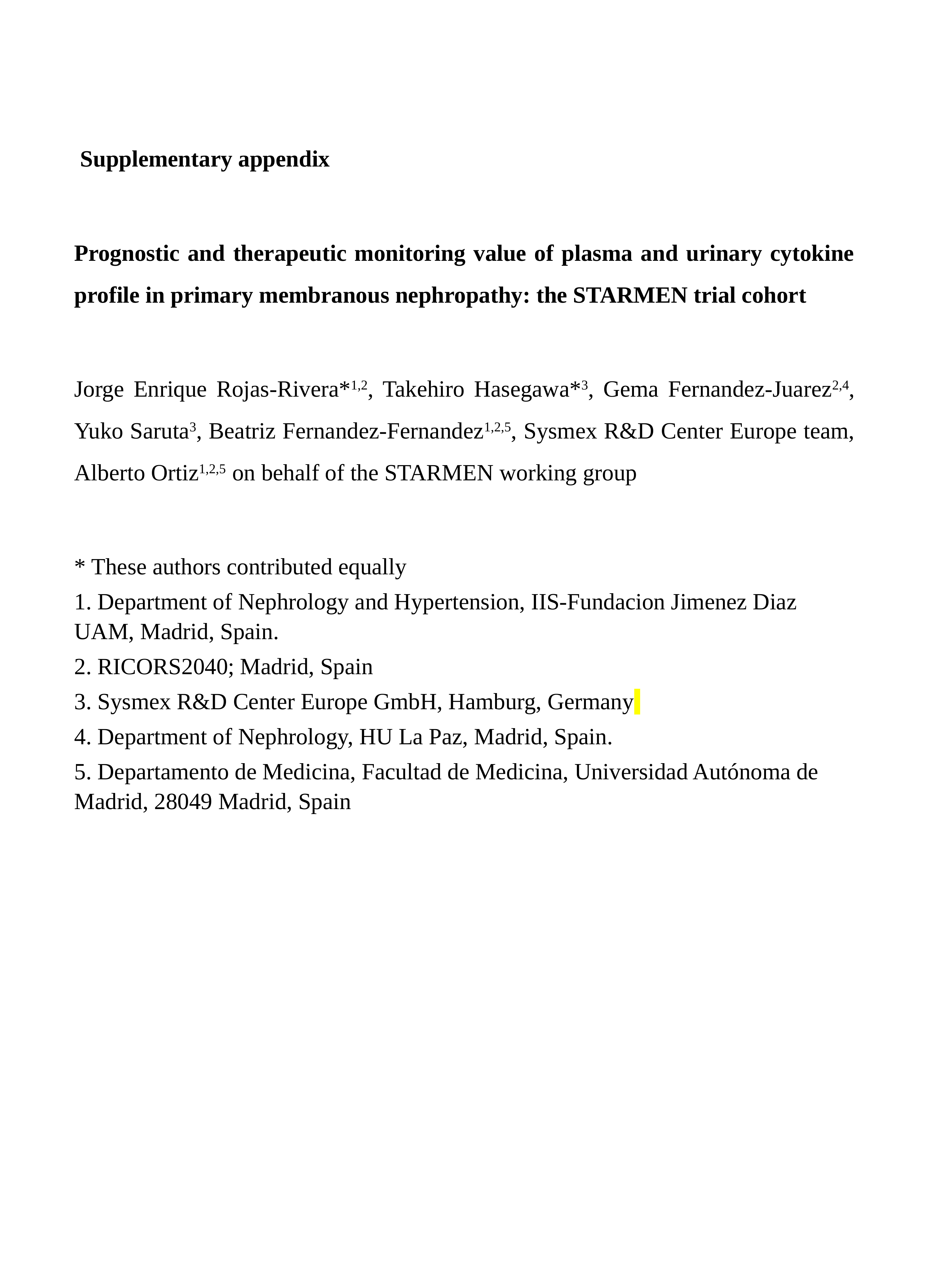

Supplementary appendix
Prognostic and therapeutic monitoring value of plasma and urinary cytokine profile in primary membranous nephropathy: the STARMEN trial cohort
Jorge Enrique Rojas-Rivera*1,2, Takehiro Hasegawa*3, Gema Fernandez-Juarez2,4, Yuko Saruta3, Beatriz Fernandez-Fernandez1,2,5, Sysmex R&D Center Europe team, Alberto Ortiz1,2,5  on behalf of the STARMEN working group
* These authors contributed equally
1. Department of Nephrology and Hypertension, IIS-Fundacion Jimenez Diaz UAM, Madrid, Spain.
2. RICORS2040; Madrid, Spain
3. Sysmex R&D Center Europe GmbH, Hamburg, Germany
4. Department of Nephrology, HU La Paz, Madrid, Spain.
5. Departamento de Medicina, Facultad de Medicina, Universidad Autónoma de Madrid, 28049 Madrid, Spain

## Slide 2
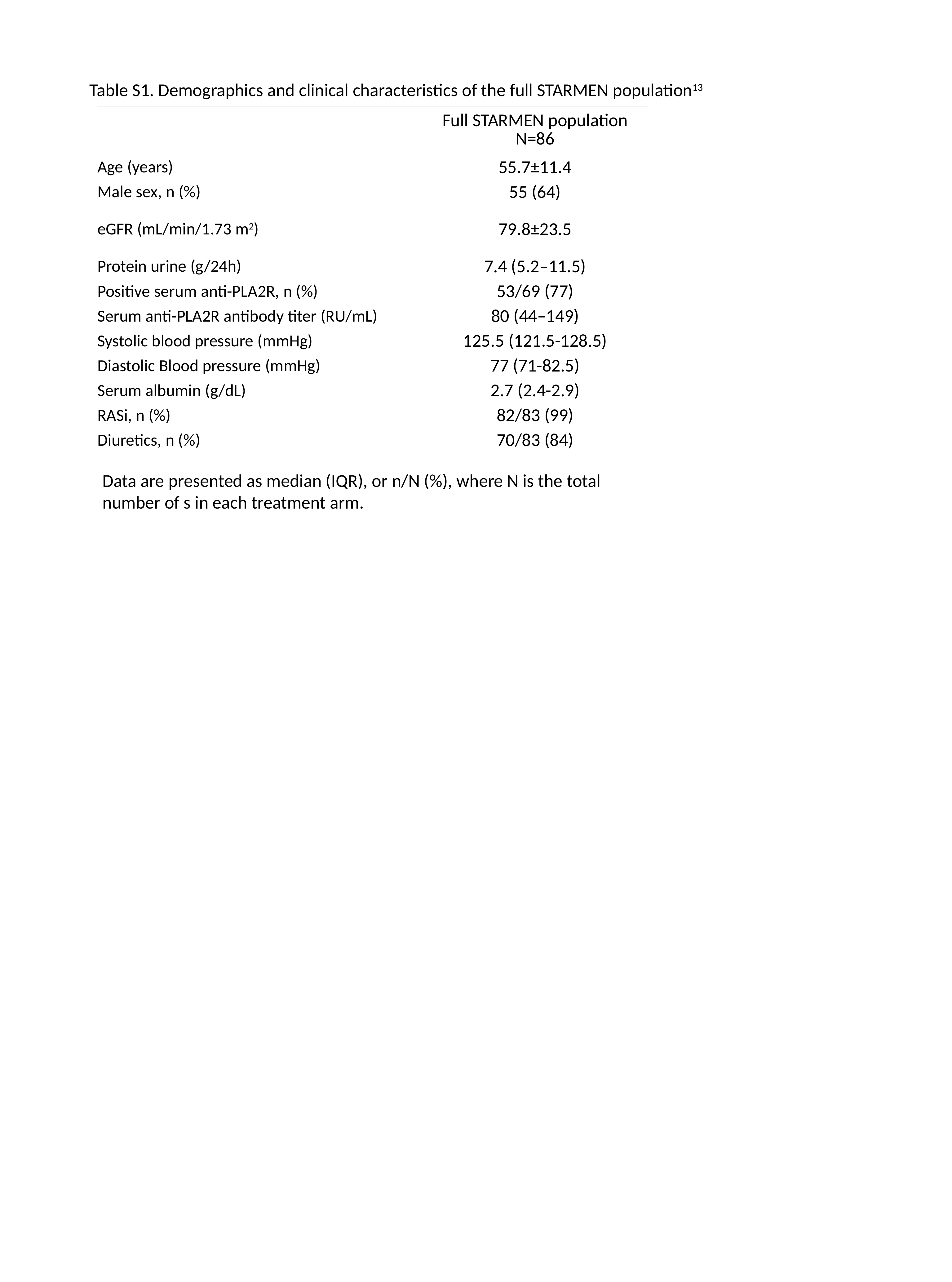

Table S1. Demographics and clinical characteristics of the full STARMEN population13
| | Full STARMEN population N=86 | |
| --- | --- | --- |
| Age (years) | 55.7±11.4 | |
| Male sex, n (%) | 55 (64) | |
| eGFR (mL/min/1.73 m2) | 79.8±23.5 | |
| | | |
| Protein urine (g/24h) | 7.4 (5.2–11.5) | |
| Positive serum anti-PLA2R, n (%) | 53/69 (77) | |
| Serum anti-PLA2R antibody titer (RU/mL) | 80 (44–149) | |
| Systolic blood pressure (mmHg) | 125.5 (121.5-128.5) | |
| Diastolic Blood pressure (mmHg) | 77 (71-82.5) | |
| Serum albumin (g/dL) | 2.7 (2.4-2.9) | |
| RASi, n (%) | 82/83 (99) | |
| Diuretics, n (%) | 70/83 (84) | |
Data are presented as median (IQR), or n/N (%), where N is the total number of s in each treatment arm.

## Slide 3
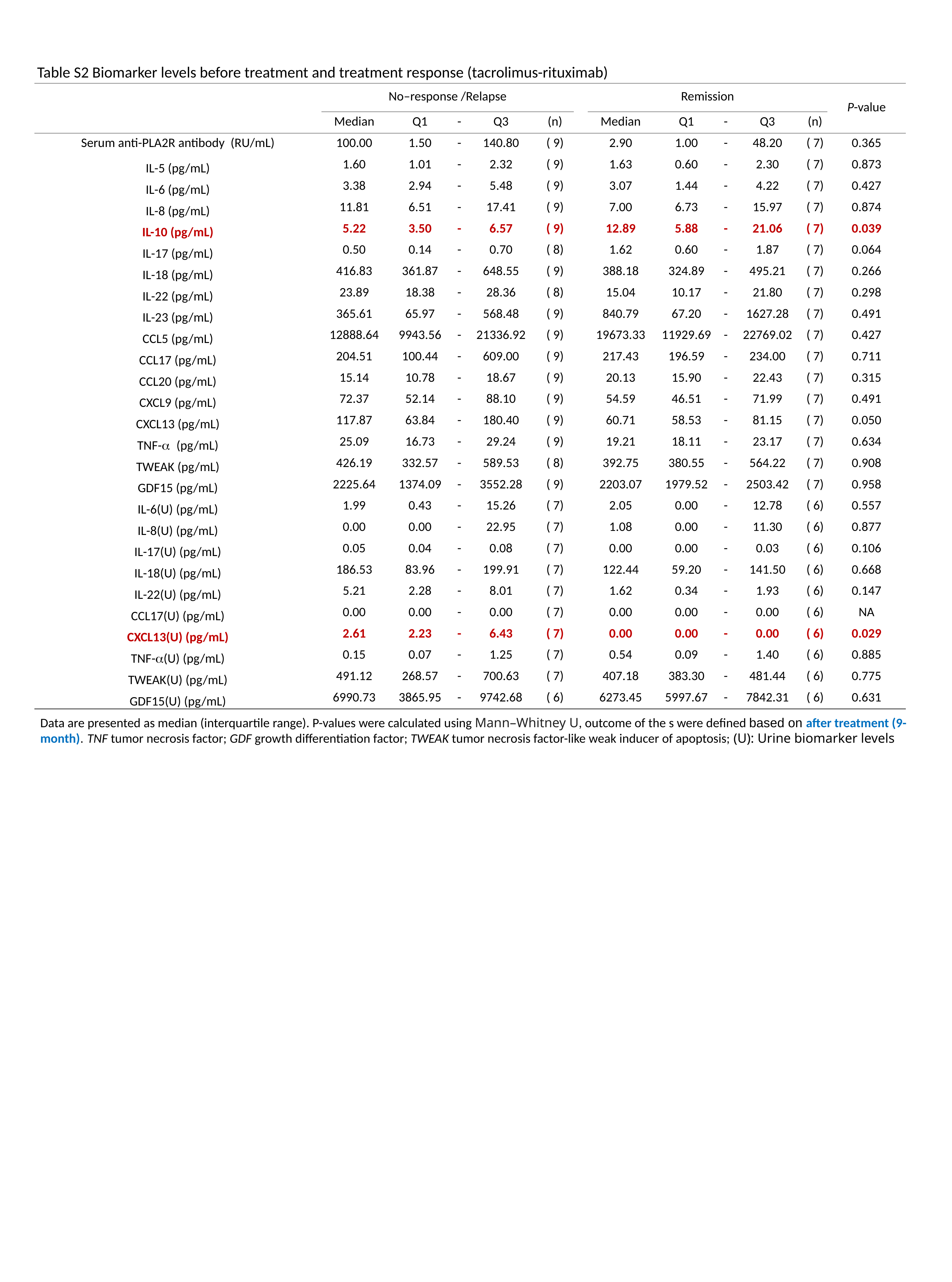

Table S2 Biomarker levels before treatment and treatment response (tacrolimus-rituximab)
| | No–response /Relapse | | | | | | Remission | | | | | P-value |
| --- | --- | --- | --- | --- | --- | --- | --- | --- | --- | --- | --- | --- |
| | Median | Q1 | - | Q3 | (n) | | Median | Q1 | - | Q3 | (n) | |
| Serum anti-PLA2R antibody (RU/mL) | 100.00 | 1.50 | - | 140.80 | ( 9) | | 2.90 | 1.00 | - | 48.20 | ( 7) | 0.365 |
| IL-5 (pg/mL) | 1.60 | 1.01 | - | 2.32 | ( 9) | | 1.63 | 0.60 | - | 2.30 | ( 7) | 0.873 |
| IL-6 (pg/mL) | 3.38 | 2.94 | - | 5.48 | ( 9) | | 3.07 | 1.44 | - | 4.22 | ( 7) | 0.427 |
| IL-8 (pg/mL) | 11.81 | 6.51 | - | 17.41 | ( 9) | | 7.00 | 6.73 | - | 15.97 | ( 7) | 0.874 |
| IL-10 (pg/mL) | 5.22 | 3.50 | - | 6.57 | ( 9) | | 12.89 | 5.88 | - | 21.06 | ( 7) | 0.039 |
| IL-17 (pg/mL) | 0.50 | 0.14 | - | 0.70 | ( 8) | | 1.62 | 0.60 | - | 1.87 | ( 7) | 0.064 |
| IL-18 (pg/mL) | 416.83 | 361.87 | - | 648.55 | ( 9) | | 388.18 | 324.89 | - | 495.21 | ( 7) | 0.266 |
| IL-22 (pg/mL) | 23.89 | 18.38 | - | 28.36 | ( 8) | | 15.04 | 10.17 | - | 21.80 | ( 7) | 0.298 |
| IL-23 (pg/mL) | 365.61 | 65.97 | - | 568.48 | ( 9) | | 840.79 | 67.20 | - | 1627.28 | ( 7) | 0.491 |
| CCL5 (pg/mL) | 12888.64 | 9943.56 | - | 21336.92 | ( 9) | | 19673.33 | 11929.69 | - | 22769.02 | ( 7) | 0.427 |
| CCL17 (pg/mL) | 204.51 | 100.44 | - | 609.00 | ( 9) | | 217.43 | 196.59 | - | 234.00 | ( 7) | 0.711 |
| CCL20 (pg/mL) | 15.14 | 10.78 | - | 18.67 | ( 9) | | 20.13 | 15.90 | - | 22.43 | ( 7) | 0.315 |
| CXCL9 (pg/mL) | 72.37 | 52.14 | - | 88.10 | ( 9) | | 54.59 | 46.51 | - | 71.99 | ( 7) | 0.491 |
| CXCL13 (pg/mL) | 117.87 | 63.84 | - | 180.40 | ( 9) | | 60.71 | 58.53 | - | 81.15 | ( 7) | 0.050 |
| TNF-a (pg/mL) | 25.09 | 16.73 | - | 29.24 | ( 9) | | 19.21 | 18.11 | - | 23.17 | ( 7) | 0.634 |
| TWEAK (pg/mL) | 426.19 | 332.57 | - | 589.53 | ( 8) | | 392.75 | 380.55 | - | 564.22 | ( 7) | 0.908 |
| GDF15 (pg/mL) | 2225.64 | 1374.09 | - | 3552.28 | ( 9) | | 2203.07 | 1979.52 | - | 2503.42 | ( 7) | 0.958 |
| IL-6(U) (pg/mL) | 1.99 | 0.43 | - | 15.26 | ( 7) | | 2.05 | 0.00 | - | 12.78 | ( 6) | 0.557 |
| IL-8(U) (pg/mL) | 0.00 | 0.00 | - | 22.95 | ( 7) | | 1.08 | 0.00 | - | 11.30 | ( 6) | 0.877 |
| IL-17(U) (pg/mL) | 0.05 | 0.04 | - | 0.08 | ( 7) | | 0.00 | 0.00 | - | 0.03 | ( 6) | 0.106 |
| IL-18(U) (pg/mL) | 186.53 | 83.96 | - | 199.91 | ( 7) | | 122.44 | 59.20 | - | 141.50 | ( 6) | 0.668 |
| IL-22(U) (pg/mL) | 5.21 | 2.28 | - | 8.01 | ( 7) | | 1.62 | 0.34 | - | 1.93 | ( 6) | 0.147 |
| CCL17(U) (pg/mL) | 0.00 | 0.00 | - | 0.00 | ( 7) | | 0.00 | 0.00 | - | 0.00 | ( 6) | NA |
| CXCL13(U) (pg/mL) | 2.61 | 2.23 | - | 6.43 | ( 7) | | 0.00 | 0.00 | - | 0.00 | ( 6) | 0.029 |
| TNF-a(U) (pg/mL) | 0.15 | 0.07 | - | 1.25 | ( 7) | | 0.54 | 0.09 | - | 1.40 | ( 6) | 0.885 |
| TWEAK(U) (pg/mL) | 491.12 | 268.57 | - | 700.63 | ( 7) | | 407.18 | 383.30 | - | 481.44 | ( 6) | 0.775 |
| GDF15(U) (pg/mL) | 6990.73 | 3865.95 | - | 9742.68 | ( 6) | | 6273.45 | 5997.67 | - | 7842.31 | ( 6) | 0.631 |
Data are presented as median (interquartile range). P-values were calculated using Mann–Whitney U, outcome of the s were defined based on after treatment (9-month). TNF tumor necrosis factor; GDF growth differentiation factor; TWEAK tumor necrosis factor-like weak inducer of apoptosis; (U): Urine biomarker levels

## Slide 4
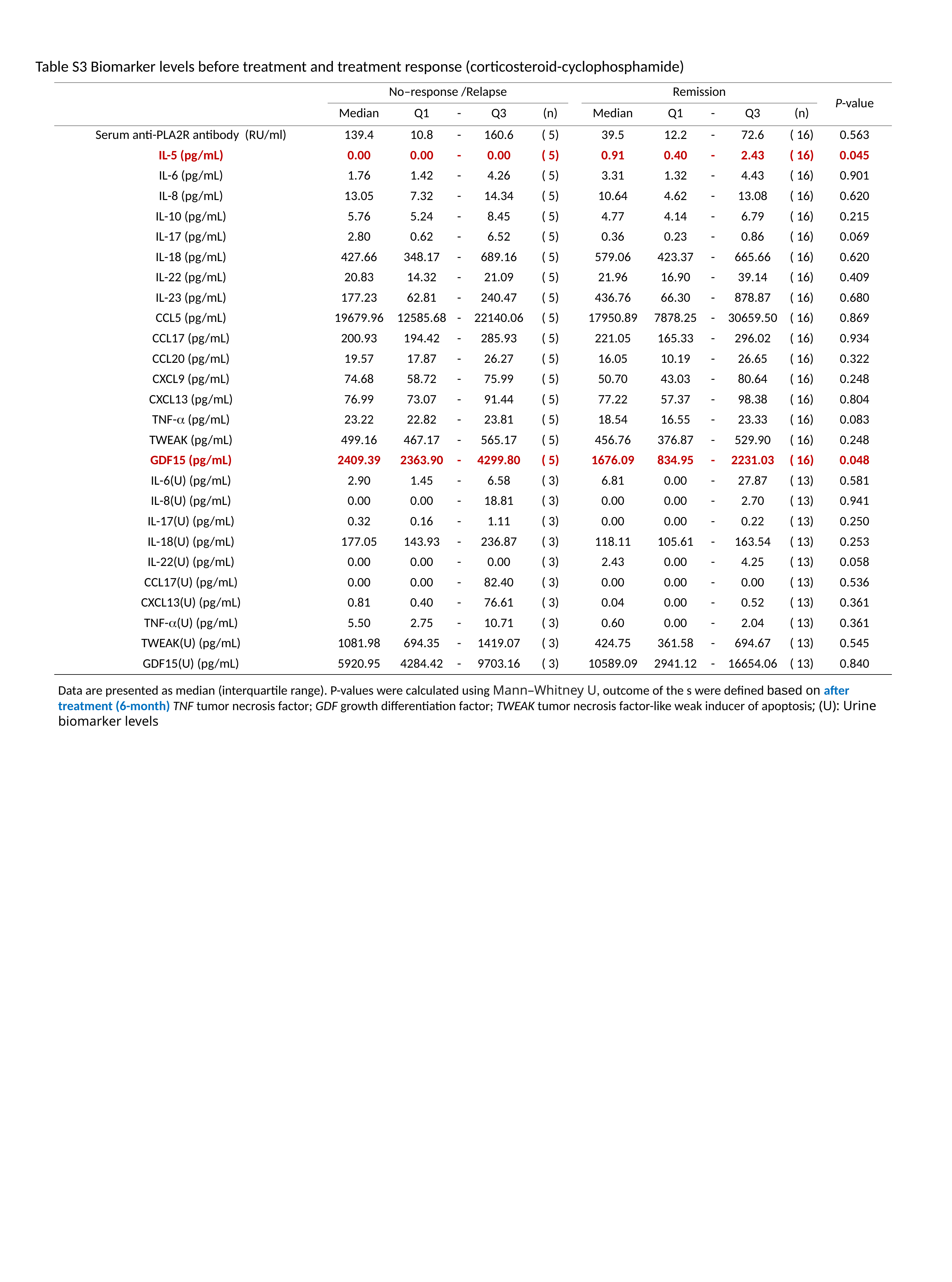

Table S3 Biomarker levels before treatment and treatment response (corticosteroid-cyclophosphamide)
| | No–response /Relapse | | | | | | Remission | | | | | P-value |
| --- | --- | --- | --- | --- | --- | --- | --- | --- | --- | --- | --- | --- |
| | Median | Q1 | - | Q3 | (n) | | Median | Q1 | - | Q3 | (n) | |
| Serum anti-PLA2R antibody (RU/ml) | 139.4 | 10.8 | - | 160.6 | ( 5) | | 39.5 | 12.2 | - | 72.6 | ( 16) | 0.563 |
| IL-5 (pg/mL) | 0.00 | 0.00 | - | 0.00 | ( 5) | | 0.91 | 0.40 | - | 2.43 | ( 16) | 0.045 |
| IL-6 (pg/mL) | 1.76 | 1.42 | - | 4.26 | ( 5) | | 3.31 | 1.32 | - | 4.43 | ( 16) | 0.901 |
| IL-8 (pg/mL) | 13.05 | 7.32 | - | 14.34 | ( 5) | | 10.64 | 4.62 | - | 13.08 | ( 16) | 0.620 |
| IL-10 (pg/mL) | 5.76 | 5.24 | - | 8.45 | ( 5) | | 4.77 | 4.14 | - | 6.79 | ( 16) | 0.215 |
| IL-17 (pg/mL) | 2.80 | 0.62 | - | 6.52 | ( 5) | | 0.36 | 0.23 | - | 0.86 | ( 16) | 0.069 |
| IL-18 (pg/mL) | 427.66 | 348.17 | - | 689.16 | ( 5) | | 579.06 | 423.37 | - | 665.66 | ( 16) | 0.620 |
| IL-22 (pg/mL) | 20.83 | 14.32 | - | 21.09 | ( 5) | | 21.96 | 16.90 | - | 39.14 | ( 16) | 0.409 |
| IL-23 (pg/mL) | 177.23 | 62.81 | - | 240.47 | ( 5) | | 436.76 | 66.30 | - | 878.87 | ( 16) | 0.680 |
| CCL5 (pg/mL) | 19679.96 | 12585.68 | - | 22140.06 | ( 5) | | 17950.89 | 7878.25 | - | 30659.50 | ( 16) | 0.869 |
| CCL17 (pg/mL) | 200.93 | 194.42 | - | 285.93 | ( 5) | | 221.05 | 165.33 | - | 296.02 | ( 16) | 0.934 |
| CCL20 (pg/mL) | 19.57 | 17.87 | - | 26.27 | ( 5) | | 16.05 | 10.19 | - | 26.65 | ( 16) | 0.322 |
| CXCL9 (pg/mL) | 74.68 | 58.72 | - | 75.99 | ( 5) | | 50.70 | 43.03 | - | 80.64 | ( 16) | 0.248 |
| CXCL13 (pg/mL) | 76.99 | 73.07 | - | 91.44 | ( 5) | | 77.22 | 57.37 | - | 98.38 | ( 16) | 0.804 |
| TNF-a (pg/mL) | 23.22 | 22.82 | - | 23.81 | ( 5) | | 18.54 | 16.55 | - | 23.33 | ( 16) | 0.083 |
| TWEAK (pg/mL) | 499.16 | 467.17 | - | 565.17 | ( 5) | | 456.76 | 376.87 | - | 529.90 | ( 16) | 0.248 |
| GDF15 (pg/mL) | 2409.39 | 2363.90 | - | 4299.80 | ( 5) | | 1676.09 | 834.95 | - | 2231.03 | ( 16) | 0.048 |
| IL-6(U) (pg/mL) | 2.90 | 1.45 | - | 6.58 | ( 3) | | 6.81 | 0.00 | - | 27.87 | ( 13) | 0.581 |
| IL-8(U) (pg/mL) | 0.00 | 0.00 | - | 18.81 | ( 3) | | 0.00 | 0.00 | - | 2.70 | ( 13) | 0.941 |
| IL-17(U) (pg/mL) | 0.32 | 0.16 | - | 1.11 | ( 3) | | 0.00 | 0.00 | - | 0.22 | ( 13) | 0.250 |
| IL-18(U) (pg/mL) | 177.05 | 143.93 | - | 236.87 | ( 3) | | 118.11 | 105.61 | - | 163.54 | ( 13) | 0.253 |
| IL-22(U) (pg/mL) | 0.00 | 0.00 | - | 0.00 | ( 3) | | 2.43 | 0.00 | - | 4.25 | ( 13) | 0.058 |
| CCL17(U) (pg/mL) | 0.00 | 0.00 | - | 82.40 | ( 3) | | 0.00 | 0.00 | - | 0.00 | ( 13) | 0.536 |
| CXCL13(U) (pg/mL) | 0.81 | 0.40 | - | 76.61 | ( 3) | | 0.04 | 0.00 | - | 0.52 | ( 13) | 0.361 |
| TNF-a(U) (pg/mL) | 5.50 | 2.75 | - | 10.71 | ( 3) | | 0.60 | 0.00 | - | 2.04 | ( 13) | 0.361 |
| TWEAK(U) (pg/mL) | 1081.98 | 694.35 | - | 1419.07 | ( 3) | | 424.75 | 361.58 | - | 694.67 | ( 13) | 0.545 |
| GDF15(U) (pg/mL) | 5920.95 | 4284.42 | - | 9703.16 | ( 3) | | 10589.09 | 2941.12 | - | 16654.06 | ( 13) | 0.840 |
Data are presented as median (interquartile range). P-values were calculated using Mann–Whitney U, outcome of the s were defined based on after treatment (6-month) TNF tumor necrosis factor; GDF growth differentiation factor; TWEAK tumor necrosis factor-like weak inducer of apoptosis; (U): Urine biomarker levels

## Slide 5
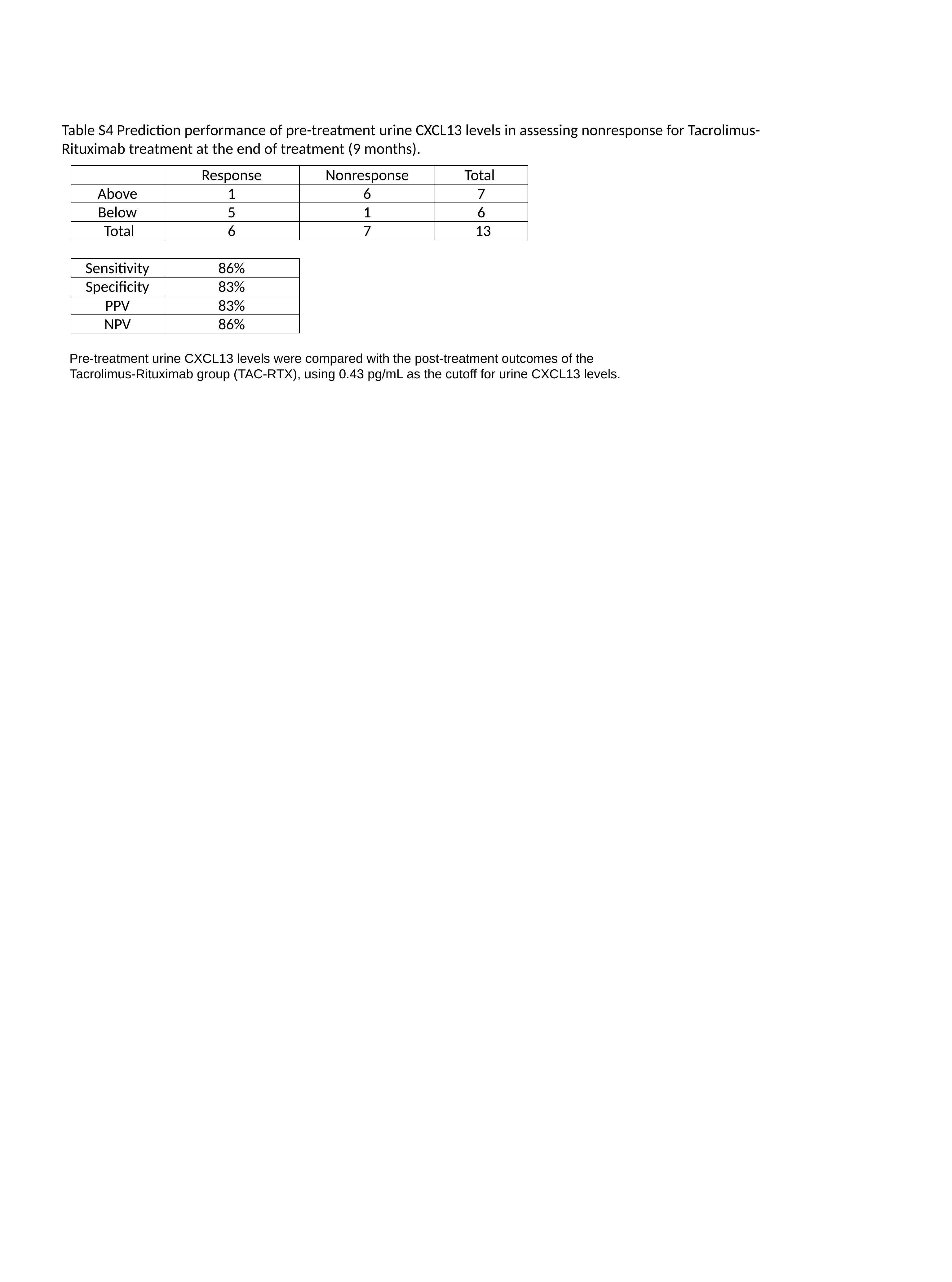

Table S4 Prediction performance of pre-treatment urine CXCL13 levels in assessing nonresponse for Tacrolimus-Rituximab treatment at the end of treatment (9 months).
| | Response | Nonresponse | Total |
| --- | --- | --- | --- |
| Above | 1 | 6 | 7 |
| Below | 5 | 1 | 6 |
| Total | 6 | 7 | 13 |
| | | | |
| Sensitivity | 86% | | |
| Specificity | 83% | | |
| PPV | 83% | | |
| NPV | 86% | | |
Pre-treatment urine CXCL13 levels were compared with the post-treatment outcomes of the Tacrolimus-Rituximab group (TAC-RTX), using 0.43 pg/mL as the cutoff for urine CXCL13 levels.

## Slide 6
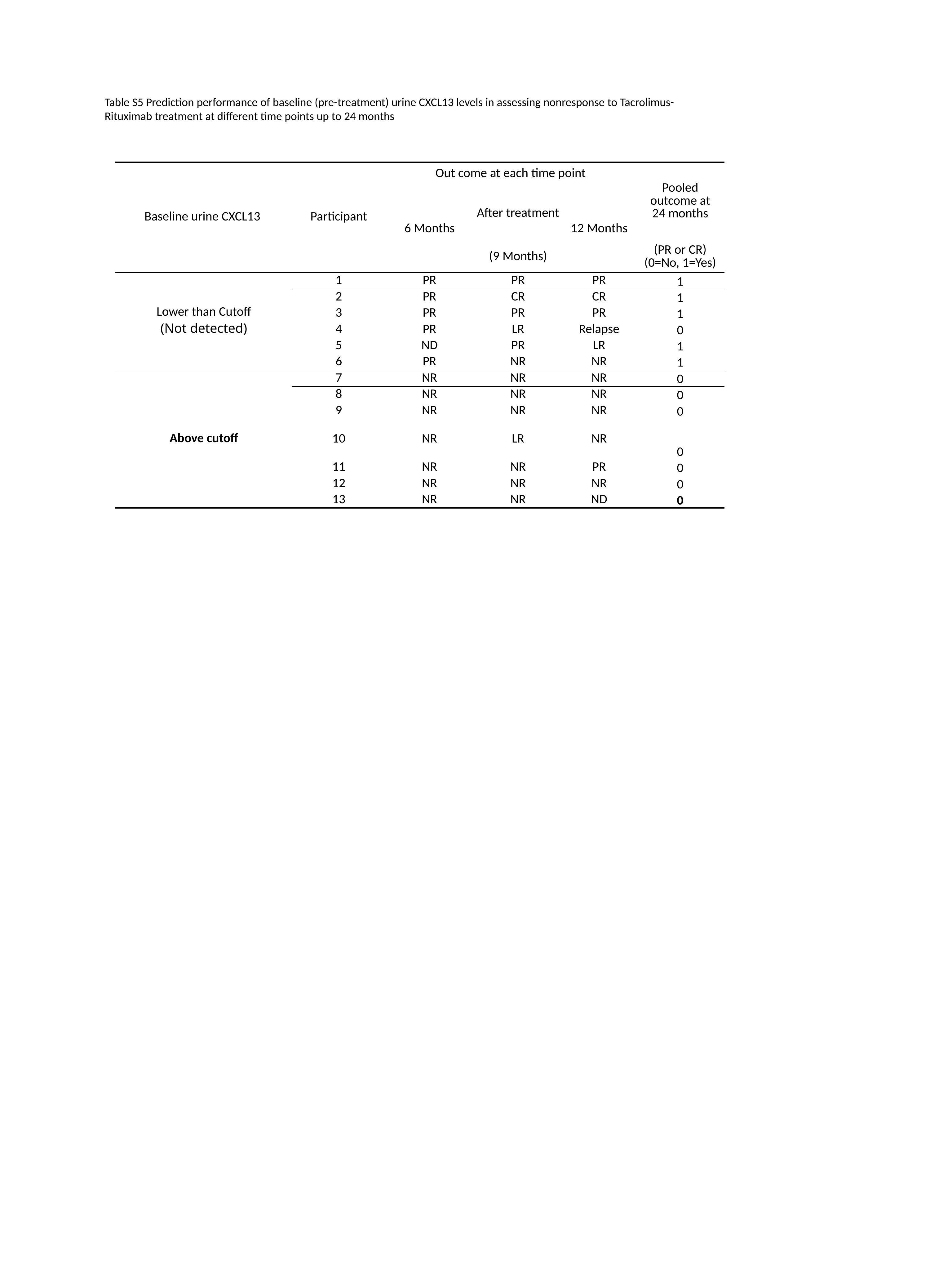

Table S5 Prediction performance of baseline (pre-treatment) urine CXCL13 levels in assessing nonresponse to Tacrolimus-Rituximab treatment at different time points up to 24 months
| Baseline urine CXCL13 | Participant | Out come at each time point | | | Pooled outcome at 24 months |
| --- | --- | --- | --- | --- | --- |
| | | 6 Months | After treatment | 12 Months | |
| | | | (9 Months) | | (PR or CR) (0=No, 1=Yes) |
| Lower than Cutoff (Not detected) | 1 | PR | PR | PR | 1 |
| | 2 | PR | CR | CR | 1 |
| | 3 | PR | PR | PR | 1 |
| | 4 | PR | LR | Relapse | 0 |
| | 5 | ND | PR | LR | 1 |
| | 6 | PR | NR | NR | 1 |
| Above cutoff | 7 | NR | NR | NR | 0 |
| | 8 | NR | NR | NR | 0 |
| | 9 | NR | NR | NR | 0 |
| | 10 | NR | LR | NR | 0 |
| | 11 | NR | NR | PR | 0 |
| | 12 | NR | NR | NR | 0 |
| | 13 | NR | NR | ND | 0 |

## Slide 7
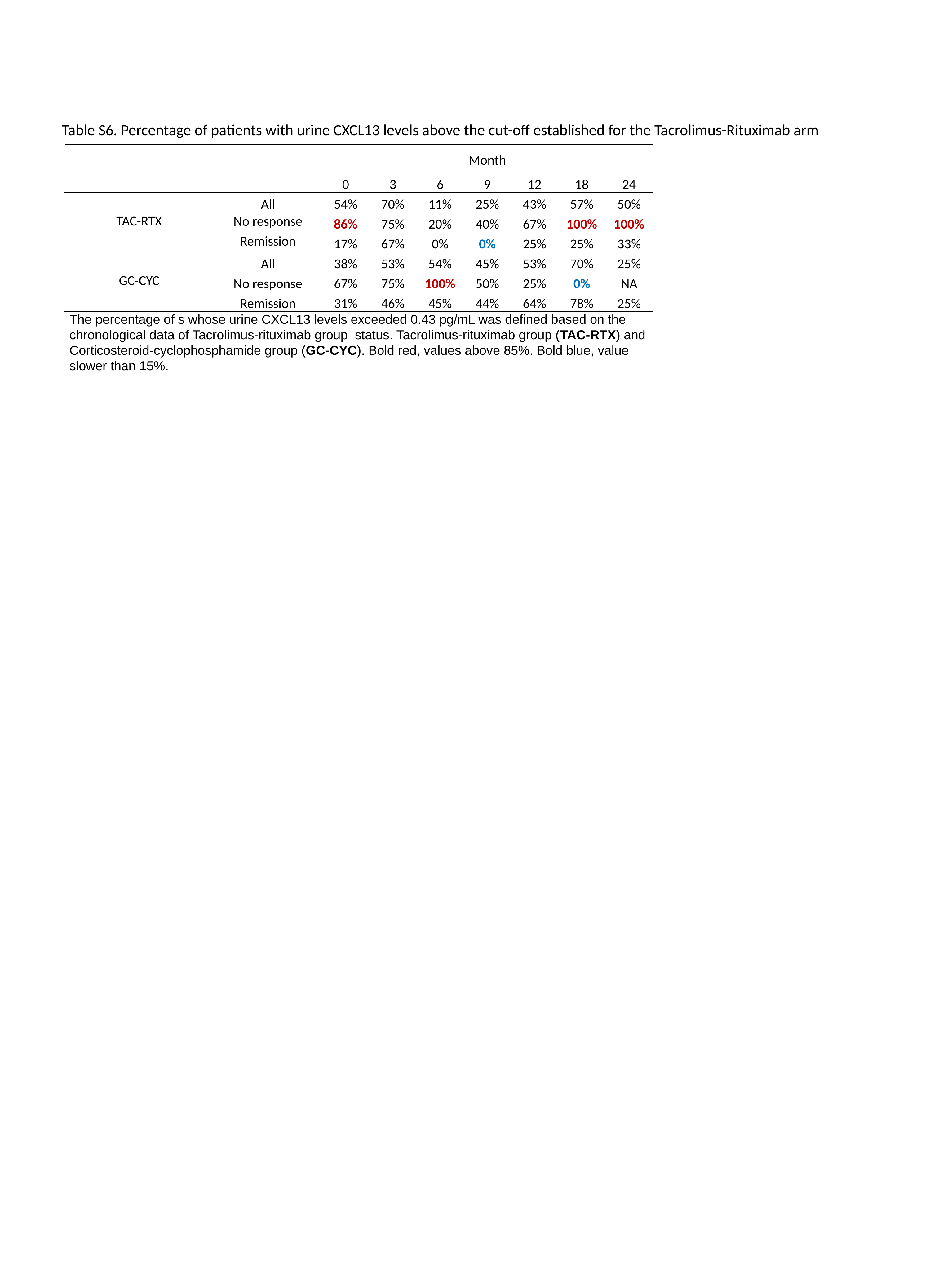

Table S6. Percentage of patients with urine CXCL13 levels above the cut-off established for the Tacrolimus-Rituximab arm
| | | Month | | | | | | |
| --- | --- | --- | --- | --- | --- | --- | --- | --- |
| | | 0 | 3 | 6 | 9 | 12 | 18 | 24 |
| TAC-RTX | All | 54% | 70% | 11% | 25% | 43% | 57% | 50% |
| | No response | 86% | 75% | 20% | 40% | 67% | 100% | 100% |
| | Remission | 17% | 67% | 0% | 0% | 25% | 25% | 33% |
| GC-CYC | All | 38% | 53% | 54% | 45% | 53% | 70% | 25% |
| | No response | 67% | 75% | 100% | 50% | 25% | 0% | NA |
| | Remission | 31% | 46% | 45% | 44% | 64% | 78% | 25% |
The percentage of s whose urine CXCL13 levels exceeded 0.43 pg/mL was defined based on the chronological data of Tacrolimus-rituximab group status. Tacrolimus-rituximab group (TAC-RTX) and Corticosteroid-cyclophosphamide group (GC-CYC). Bold red, values above 85%. Bold blue, value slower than 15%.

## Slide 8
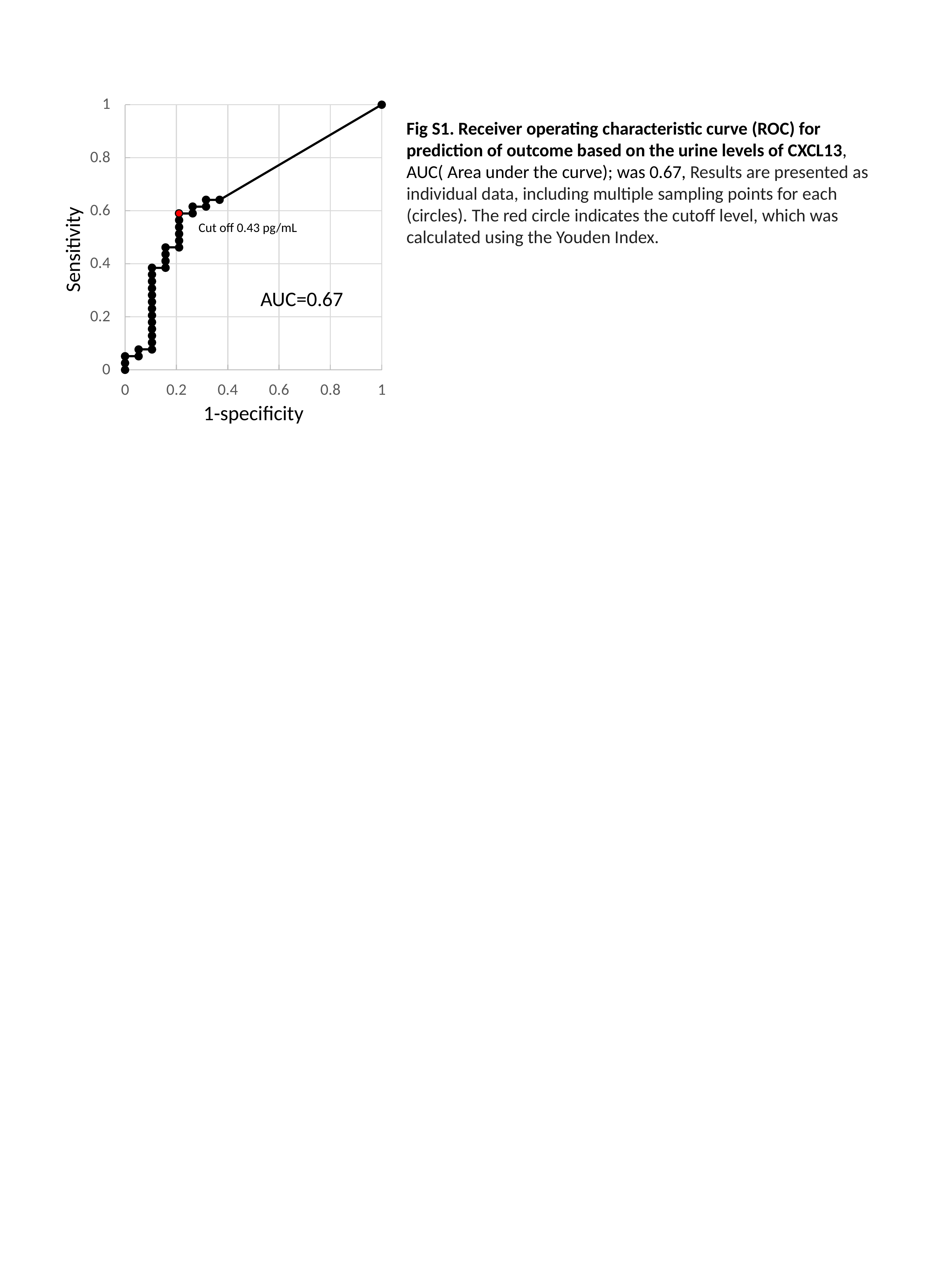

Fig S1. Receiver operating characteristic curve (ROC) for prediction of outcome based on the urine levels of CXCL13, AUC( Area under the curve); was 0.67, Results are presented as individual data, including multiple sampling points for each (circles). The red circle indicates the cutoff level, which was calculated using the Youden Index.
Cut off 0.43 pg/mL
Sensitivity
AUC=0.67
1-specificity

## Slide 9
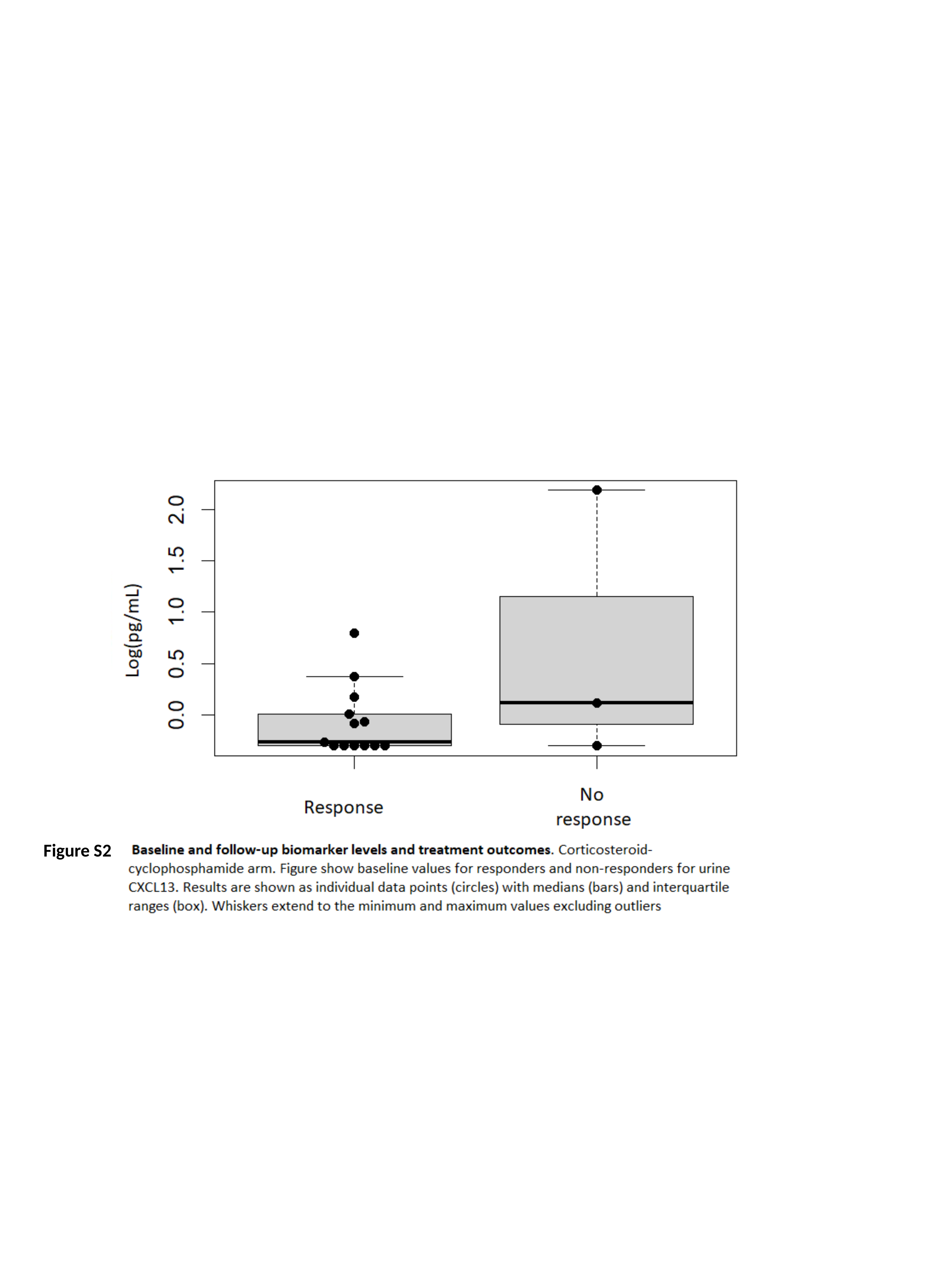

Figure S2

## Slide 10
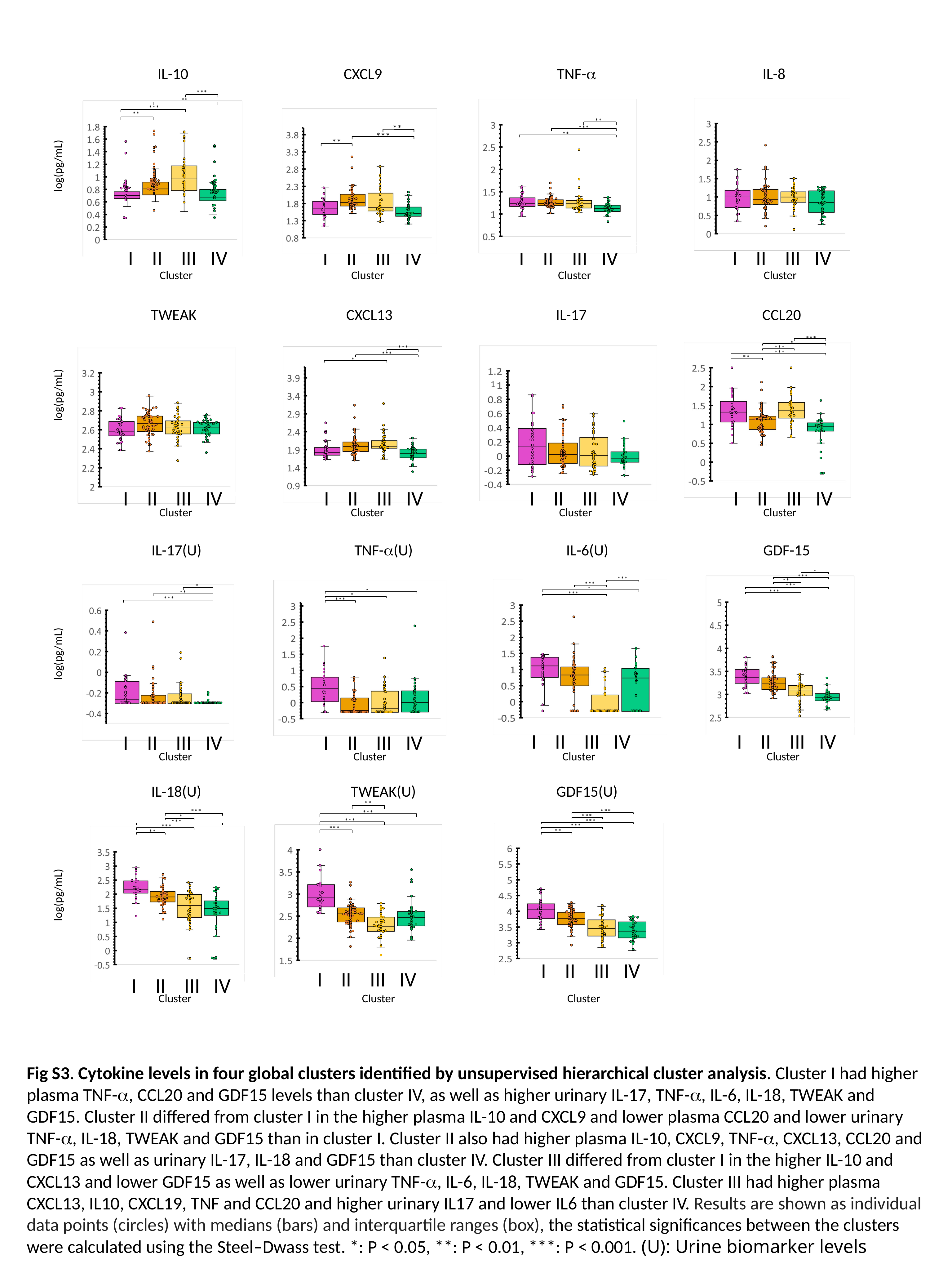

IL-10
CXCL9
TNF-a
IL-8
***
**
***
**
5
10
15
5
10
15
2
4
6
8
10
12
14
0
5
10
15
**
***
**
**
***
**
log(pg/mL)
I II III IV
I II III IV
I II III IV
I II III IV
Cluster
Cluster
Cluster
Cluster
TWEAK
CXCL13
IL-17
CCL20
***
*
***
***
**
***
***
*
0
2
4
6
8
10
12
14
1
0
2
4
6
8
10
12
14
0
5
10
15
0
5
10
15
log(pg/mL)
I II III IV
I II III IV
I II III IV
I II III IV
Cluster
Cluster
Cluster
Cluster
IL-17(U)
TNF-a(U)
IL-6(U)
GDF-15
*
***
**
***
***
***
***
*
***
0
2
4
6
8
10
0
5
10
15
20
25
30
35
*
**
***
0
5
10
15
20
25
30
35
*
*
***
0
10
20
30
40
log(pg/mL)
I II III IV
I II III IV
I II III IV
I II III IV
Cluster
Cluster
Cluster
Cluster
IL-18(U)
TWEAK(U)
GDF15(U)
**
***
***
***
***
***
***
***
**
***
*
***
***
**
0
2
4
6
8
10
0
2
4
6
8
0
2
4
6
8
10
log(pg/mL)
10
I II III IV
I II III IV
I II III IV
Cluster
Cluster
Cluster
Fig S3. Cytokine levels in four global clusters identified by unsupervised hierarchical cluster analysis. Cluster I had higher plasma TNF-a, CCL20 and GDF15 levels than cluster IV, as well as higher urinary IL-17, TNF-a, IL-6, IL-18, TWEAK and GDF15. Cluster II differed from cluster I in the higher plasma IL-10 and CXCL9 and lower plasma CCL20 and lower urinary TNF-a, IL-18, TWEAK and GDF15 than in cluster I. Cluster II also had higher plasma IL-10, CXCL9, TNF-a, CXCL13, CCL20 and GDF15 as well as urinary IL-17, IL-18 and GDF15 than cluster IV. Cluster III differed from cluster I in the higher IL-10 and CXCL13 and lower GDF15 as well as lower urinary TNF-a, IL-6, IL-18, TWEAK and GDF15. Cluster III had higher plasma CXCL13, IL10, CXCL19, TNF and CCL20 and higher urinary IL17 and lower IL6 than cluster IV. Results are shown as individual data points (circles) with medians (bars) and interquartile ranges (box), the statistical significances between the clusters were calculated using the Steel–Dwass test. *: P < 0.05, **: P < 0.01, ***: P < 0.001. (U): Urine biomarker levels

## Slide 11
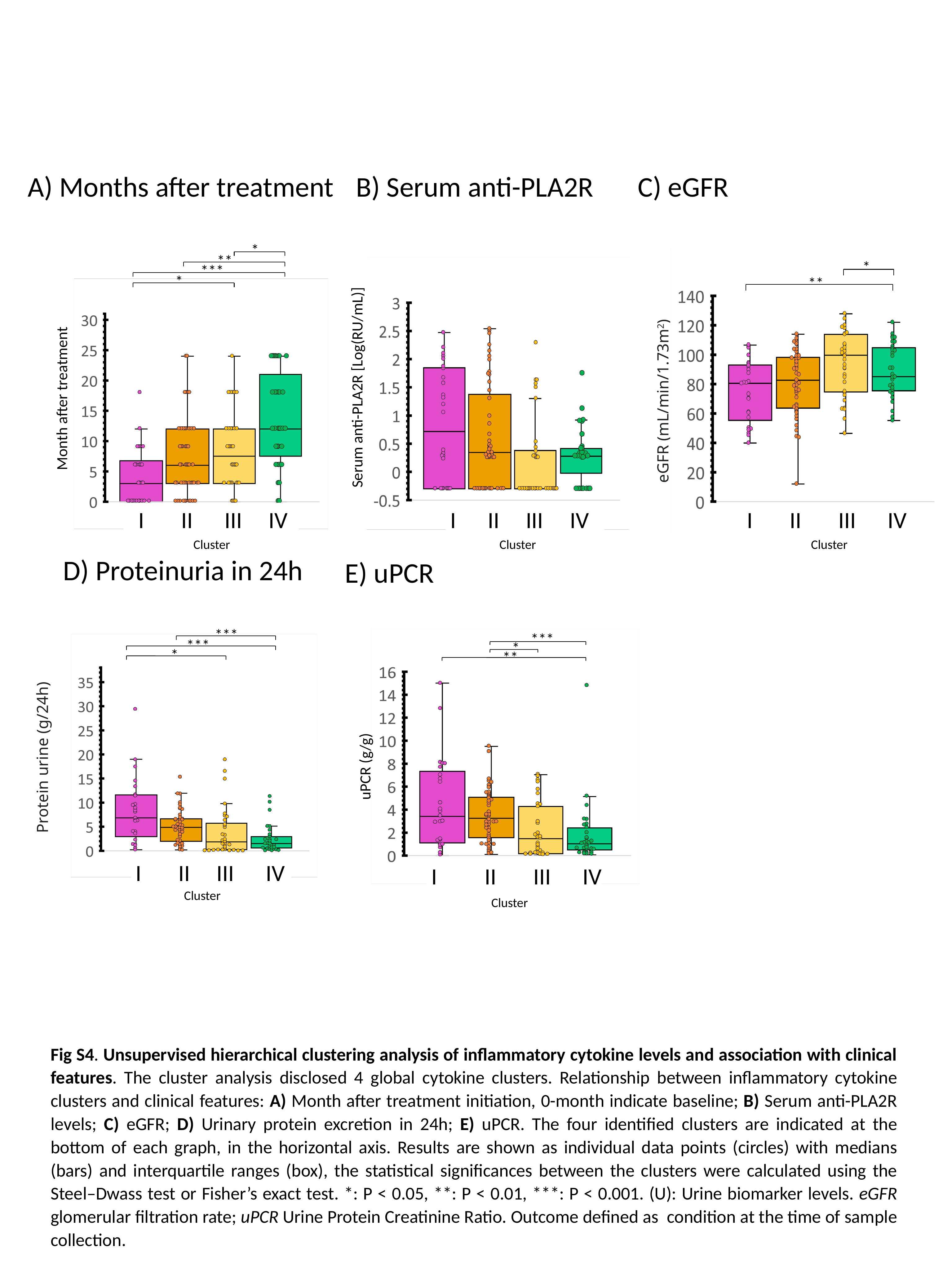

A) Months after treatment
B) Serum anti-PLA2R
C) eGFR
*
**
***
*
5
10
15
20
25
Month after treatment
0
30
 I II III IV
Cluster
0
2
4
6
8
10
12
14
*
**
eGFR (mL/min/1.73m2)
I II III IV
Cluster
0
5
10
15
20
25
30
I II III IV
Cluster
Serum anti-PLA2R [Log(RU/mL)]
D) Proteinuria in 24h
***
***
*
**
Protein urine (g/24h)
I II III IV
0
5
10
15
Cluster
E) uPCR
***
*
**
uPCR (g/g)
I II III IV
0
5
10
15
Cluster
Fig S4. Unsupervised hierarchical clustering analysis of inflammatory cytokine levels and association with clinical features. The cluster analysis disclosed 4 global cytokine clusters. Relationship between inflammatory cytokine clusters and clinical features: A) Month after treatment initiation, 0-month indicate baseline; B) Serum anti-PLA2R levels; C) eGFR; D) Urinary protein excretion in 24h; E) uPCR. The four identified clusters are indicated at the bottom of each graph, in the horizontal axis. Results are shown as individual data points (circles) with medians (bars) and interquartile ranges (box), the statistical significances between the clusters were calculated using the Steel–Dwass test or Fisher’s exact test. *: P < 0.05, **: P < 0.01, ***: P < 0.001. (U): Urine biomarker levels. eGFR glomerular filtration rate; uPCR Urine Protein Creatinine Ratio. Outcome defined as condition at the time of sample collection.

## Slide 12
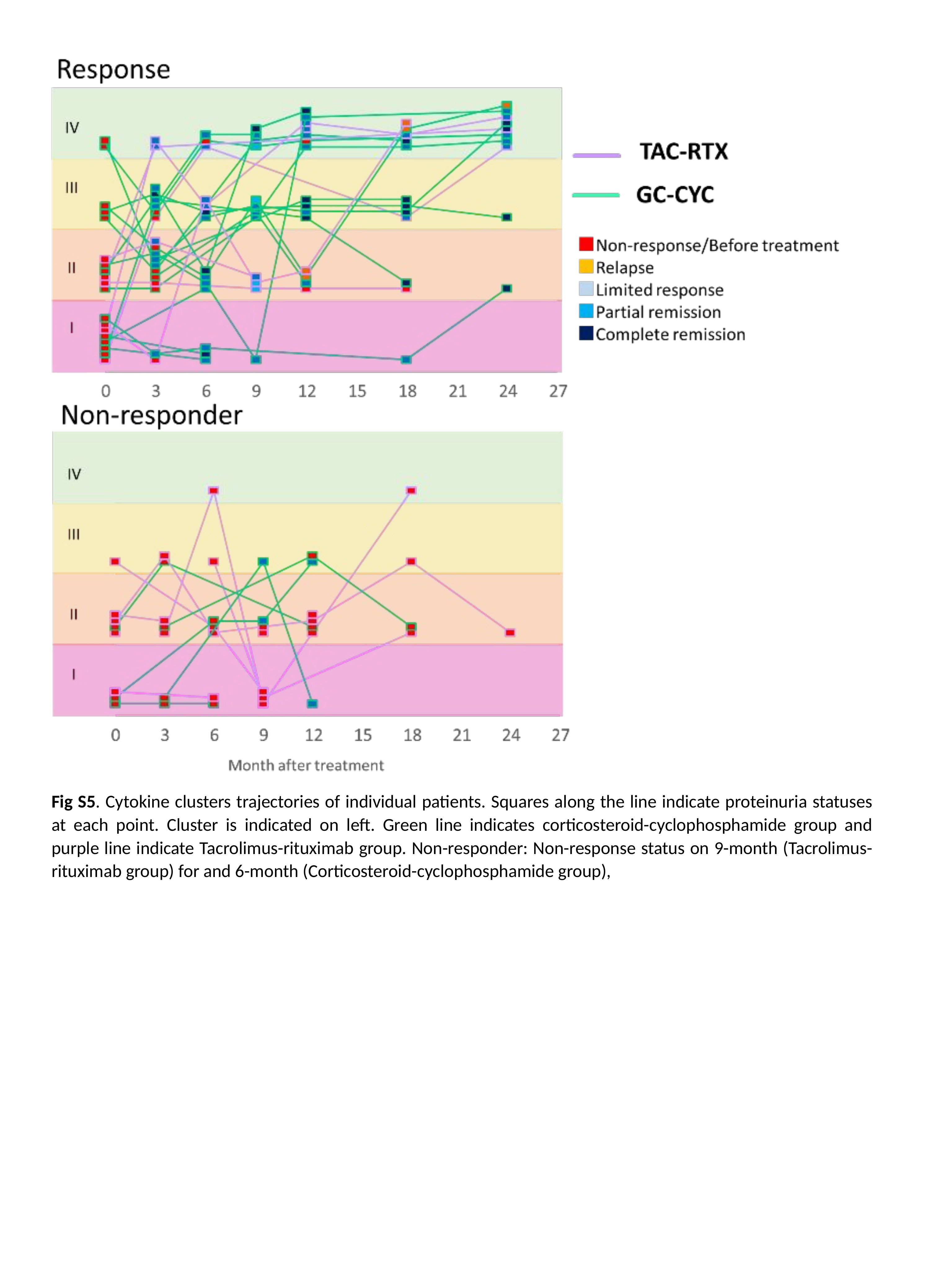

Fig S5. Cytokine clusters trajectories of individual patients. Squares along the line indicate proteinuria statuses at each point. Cluster is indicated on left. Green line indicates corticosteroid-cyclophosphamide group and purple line indicate Tacrolimus-rituximab group. Non-responder: Non-response status on 9-month (Tacrolimus-rituximab group) for and 6-month (Corticosteroid-cyclophosphamide group),

## Slide 13
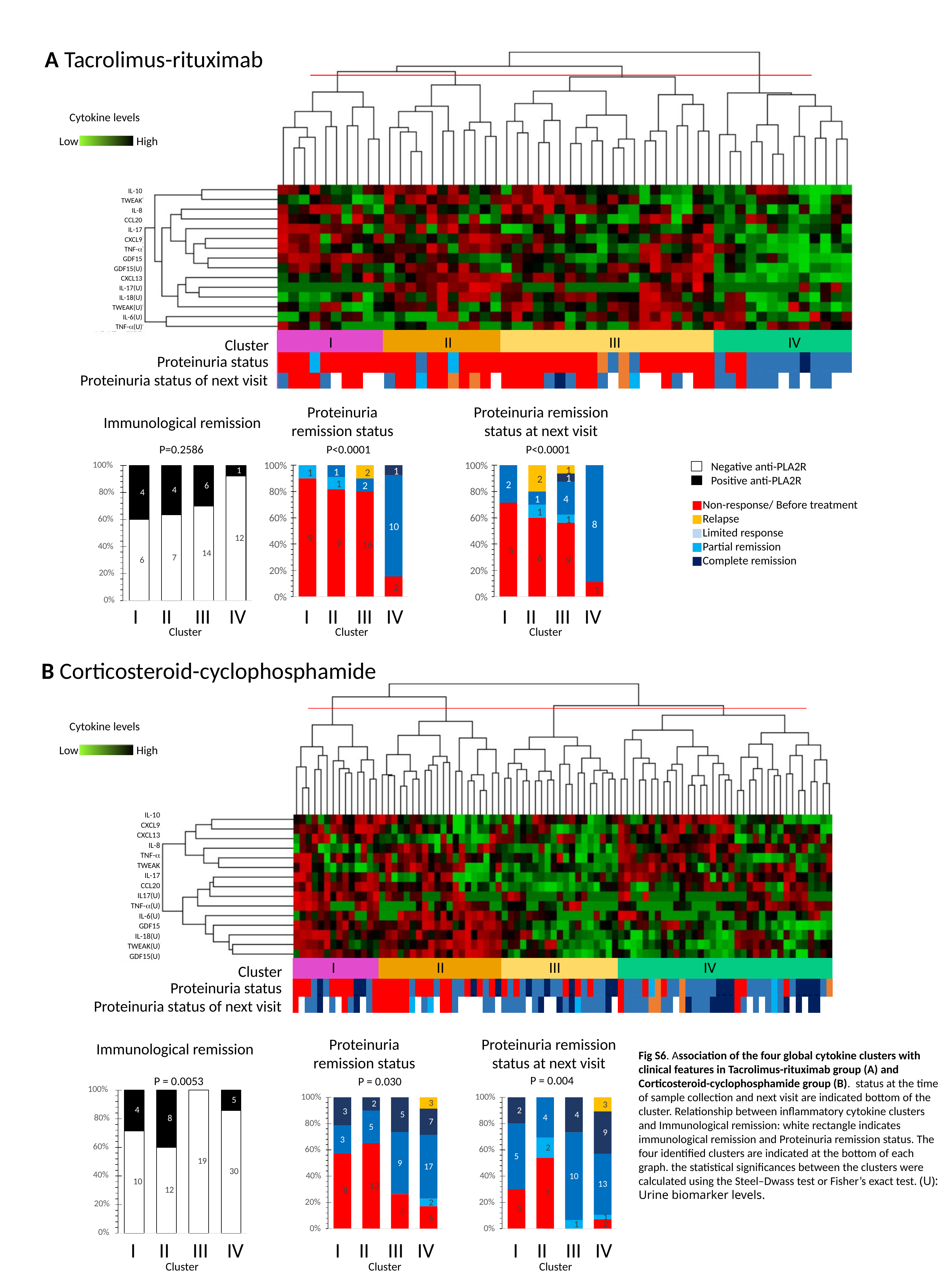

A Tacrolimus-rituximab
Cytokine levels
Low High
| IL-10 |
| --- |
| TWEAK |
| IL-8 |
| CCL20 |
| IL-17 |
| CXCL9 |
| TNF-a |
| GDF15 |
| GDF15(U) |
| CXCL13 |
| IL-17(U) |
| IL-18(U) |
| TWEAK(U) |
| IL-6(U) |
| TNF-a(U) |
I II III IV
Cluster
Proteinuria status
Proteinuria status of next visit
Proteinuria remission status
Proteinuria remission status at next visit
Immunological remission
P=0.2586
P<0.0001
P<0.0001
Negative anti-PLA2R
Positive anti-PLA2R
■Non-response/ Before treatment
■Relapse
■Limited response
■Partial remission
■Complete remission
100%
1
6
4
80%
4
60%
12
40%
14
7
6
20%
0%
I
II
III
IV
100%
1
1
1
2
1
2
80%
60%
10
9
9
40%
16
20%
2
0%
100%
1
1
2
2
80%
1
4
1
60%
1
8
40%
5
6
9
20%
1
0%
I II III IV
I II III IV
I II III IV
I
II
III
IV
I
II
III
IV
Cluster
Cluster
Cluster
B Corticosteroid-cyclophosphamide
Cluster
Proteinuria status
Proteinuria status of next visit
Cytokine levels
Low High
| IL-10 |
| --- |
| CXCL9 |
| CXCL13 |
| IL-8 |
| TNF-a |
| TWEAK |
| IL-17 |
| CCL20 |
| IL17(U) |
| TNF-a(U) |
| IL-6(U) |
| GDF15 |
| IL-18(U) |
| TWEAK(U) |
| GDF15(U) |
I II III IV
Proteinuria remission status
Proteinuria remission status at next visit
Immunological remission
Fig S6. Association of the four global cytokine clusters with clinical features in Tacrolimus-rituximab group (A) and Corticosteroid-cyclophosphamide group (B). status at the time of sample collection and next visit are indicated bottom of the cluster. Relationship between inflammatory cytokine clusters and Immunological remission: white rectangle indicates immunological remission and Proteinuria remission status. The four identified clusters are indicated at the bottom of each graph. the statistical significances between the clusters were calculated using the Steel–Dwass test or Fisher’s exact test. (U): Urine biomarker levels.
P = 0.004
P = 0.0053
P = 0.030
100%
5
4
80%
8
60%
19
30
40%
10
12
20%
0%
100%
3
2
3
5
7
80%
5
3
60%
9
17
40%
13
8
20%
2
5
6
0%
100%
3
2
4
4
80%
9
2
60%
5
40%
10
13
7
20%
3
1
2
1
0%
I II III IV
I II III IV
I II III IV
Cluster
Cluster
Cluster

## Slide 14
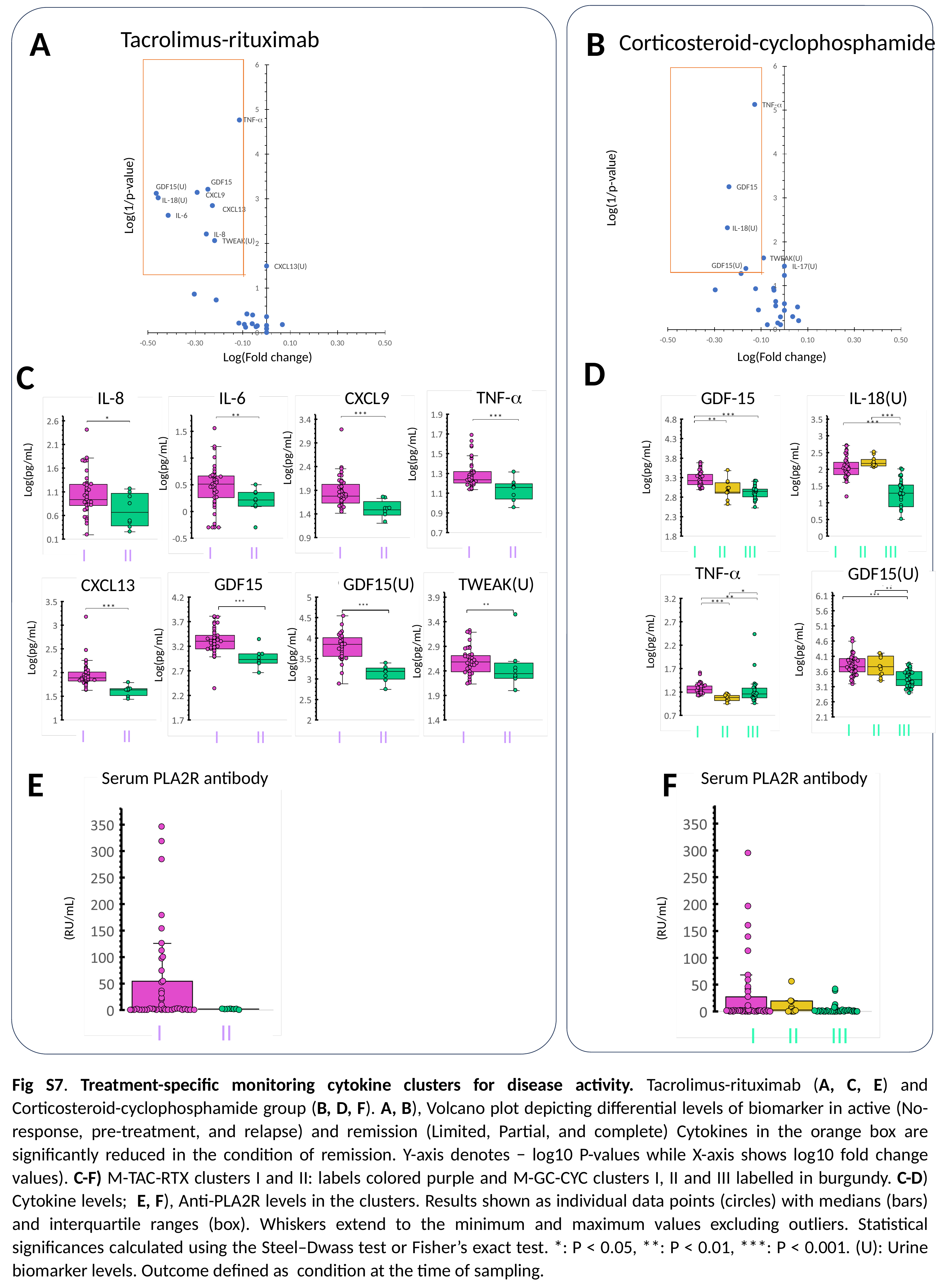

A
B
Tacrolimus-rituximab
Corticosteroid-cyclophosphamide
6
5
TNF-a
4
GDF15
GDF15(U)
CXCL9
3
IL
-
18(U)
CXCL13
IL
-
6
IL-8
TWEAK(U)
2
CXCL13(U)
1
0
-0.50
-0.30
-0.10
0.10
0.30
0.50
Log(1/p-value)
Log(Fold change)
6
TNF
-
a
5
4
Log(1/p-value)
GDF15
3
IL
-
18(U)
2
TWEAK(U)
GDF15(U)
IL-17(U)
1
0
-0.50
-0.30
-0.10
0.10
0.30
0.50
Log(Fold change)
D
C
TNF-a
IL-8
*
Log(pg/mL)
I II
I II
2
4
6
IL-6
**
Log(pg/mL)
I II
2
4
8
CXCL9
***
Log(pg/mL)
2
4
6
8
8
GDF-15
IL-18(U)
***
**
***
***
Log(pg/mL)
Log(pg/mL)
I II III
I II III
0
5
10
15
0
5
10
15
TNF-a
***
Log(pg/mL)
0
2
4
6
8
I II
I II
TNF-a
GDF15(U)
GDF15
GDF15(U)
TWEAK(U)
CXCL13
***
Log(pg/mL)
I II
2
4
6
0
5
10
15
**
***
Log(pg/mL)
I II III
0.5
1
1.5
2
2.5
2
4
6
8
*
**
***
***
**
***
Log(pg/mL)
Log(pg/mL)
Log(pg/mL)
Log(pg/mL)
I II III
0
5
10
15
I II
I II
I II
E
F
Serum PLA2R antibody
Serum PLA2R antibody
0
5
10
15
20
(RU/mL)
(RU/mL)
I II
8
I II III
Fig S7. Treatment-specific monitoring cytokine clusters for disease activity. Tacrolimus-rituximab (A, C, E) and Corticosteroid-cyclophosphamide group (B, D, F). A, B), Volcano plot depicting differential levels of biomarker in active (No-response, pre-treatment, and relapse) and remission (Limited, Partial, and complete) Cytokines in the orange box are significantly reduced in the condition of remission. Y-axis denotes − log10 P-values while X-axis shows log10 fold change values). C-F) M-TAC-RTX clusters I and II: labels colored purple and M-GC-CYC clusters I, II and III labelled in burgundy. C-D) Cytokine levels; E, F), Anti-PLA2R levels in the clusters. Results shown as individual data points (circles) with medians (bars) and interquartile ranges (box). Whiskers extend to the minimum and maximum values excluding outliers. Statistical significances calculated using the Steel–Dwass test or Fisher’s exact test. *: P < 0.05, **: P < 0.01, ***: P < 0.001. (U): Urine biomarker levels. Outcome defined as condition at the time of sampling.
